# Chrononutrition and Energy Balance: How Meal Timing and Circadian Rhythms Shape Weight Regulation and Metabolic Health

**DOI:** 10.3390/nu17132135

**Published:** 2025-06-27

**Authors:** Claudia Reytor-González, Daniel Simancas-Racines, Náthaly Mercedes Román-Galeano, Giuseppe Annunziata, Martina Galasso, Raynier Zambrano-Villacres, Ludovica Verde, Giovanna Muscogiuri, Evelyn Frias-Toral, Luigi Barrea

**Affiliations:** 1 Universidad UTE, Facultad de Ciencias de la Salud Eugenio Espejo, Centro de Investigación en Salud Pública y Epidemiología Clínica (CISPEC), Quito 170527, Ecuador; claudia.reytor@ute.edu.ec (C.R.-G.); nathalyroman0001@gmail.com (N.M.R.-G.); 2 Department of Experimental Medicine, University of Campania “Luigi Vanvitelli”, 80138 Naples, Italy; giuseppe.annunziata@unipegaso.it; 3 Unità Di Endocrinologia, Dipartimento Di Medicina Clinica E Chirurgia, Università degli Studi di Napoli Federico II, 80131 Naples, Italygiovanna.muscogiuri@unina.it (G.M.); 4 Dipartimento di Endocrinologia, Diabetologia, Andrologia e Nutrizione, Centro Italiano Per La Cura E Il Benessere del Paziente Con Obesità (C.I.B.O), AOU Federico II, Via Sergio Pansini 5, 80131 Naples, Italy; 5 Escuela de Nutrición y Dietética, Universidad Espíritu Santo, Samborondón 0901952, Ecuador; rzambranovillacres@uees.edu.ec; 6 Department of Public Health, University of Naples Federico II, Via Sergio Pansini 5, 80131 Naples, Italy; ludovica.verde@unina.it; 7 Department of Medicine, Division of Endocrinology, University of Arizona, Tucson, AZ 85724, USA; 8 Cattedra Unesco “Educazione Alla Salute E Allo Sviluppo Sostenibile”, University Federico II, 80131 Naples, Italy; 9 Escuela de Medicina, Universidad Espíritu Santo, Samborondón 0901952, Ecuador; 10 Division of Research, Texas State University, 601 University Dr, San Marcos, TX 78666, USA; 11 Dipartimento Psicologia e Scienze della Salute, Università Telematica Pegaso, Centro Direzionale Isola F2, Via Porzio, 80143 Naples, Italy

**Keywords:** chrononutrition, circadian rhythms, time-restricted eating, metabolic health, obesity, insulin sensitivity, meal timing, intermittent fasting, healthcare

## Abstract

Obesity and metabolic disorders remain major global health concerns, traditionally attributed to excessive caloric intake and poor diet quality. Recent studies emphasize that the timing of meals plays a crucial role in determining metabolic health. This review explores chrononutrition, a growing field that examines how food intake patterns interact with endogenous circadian rhythms to influence energy balance, glucose and lipid metabolism, and cardiometabolic risk. The circadian system, which includes a central clock in the suprachiasmatic nucleus and peripheral clocks in metabolic tissues, regulates physiological functions on a 24 h cycle. While light entrains the central clock, feeding schedules act as key synchronizers for peripheral clocks. Disrupting this alignment—common in modern lifestyles involving shift work or late-night eating—can impair hormonal rhythms, reduce insulin sensitivity, and promote adiposity. Evidence from clinical and preclinical studies suggests that early time-restricted eating, where food intake is confined to the morning or early afternoon, offers significant benefits for weight control, glycemic regulation, lipid profiles, and mitochondrial efficiency, even in the absence of caloric restriction. These effects are particularly relevant for populations vulnerable to circadian disruption, such as adolescents, older adults, and night-shift workers. In conclusion, aligning food intake with circadian biology represents a promising, low-cost, and modifiable strategy to improve metabolic outcomes. Integrating chrononutrition into clinical and public health strategies may enhance dietary adherence and treatment efficacy. Future large-scale studies are needed to define optimal eating windows, assess long-term sustainability, and establish population-specific chrononutritional guidelines.

## 1. Introduction

Obesity and its associated metabolic disorders, including type 2 diabetes mellitus (T2DM), cardiovascular diseases, and metabolic dysfunction-associated steatotic liver disease (MASLD), have emerged as significant public health challenges worldwide [1,2,3,4,5,6,7,8,9]. The World Health Organization (WHO) reports a tripling of global obesity rates since 1975, with over 650 million adults diagnosed with obesity in 2016 [10]. In the United States, the prevalence of obesity has remained steady at approximately 40%, while severe obesity has increased, particularly among women, reaching nearly 10% of the population [11]. This growing trend highlights the pressing need for effective approaches to address obesity and its associated metabolic disorders. Historically, dietary interventions for weight management and other numerous systemic health conditions have emphasized caloric restriction and macronutrient composition [6,12,13]. Some of these traditional approaches are the Mediterranean diet—rich in fruits, vegetables, legumes, fish, and healthy fats [14,15,16,17,18,19]—as well as the ketogenic diet (KD) and the very-low-calorie ketogenic diet (VLCKD) [20,21,22,23,24,25,26]. Both KD and VLCKD promote a state of ketosis through severe carbohydrate restriction (<50 g/day), which contributes to improvements in insulin sensitivity and metabolic parameters. The VLCKD is characterized by an energy intake of less than 800 kcal/day, with approximately 13% carbohydrates, 44% protein (1–1.5 g/kg of body weight), and 43% fat, and is primarily used for rapid weight loss and intensive metabolic intervention [23]. In contrast, the standard KD typically allows for a higher caloric intake, with a macronutrient distribution emphasizing high fat, moderate-to-low protein, and minimal carbohydrates [26]. The energy balance model posits that weight gain results from an imbalance between energy intake and expenditure regulated by brain response (internal signals to control food intake) to external signals from food [27,28]. Consequently, dietary guidelines have focused on reducing caloric intake and adjusting macronutrient ratios to promote weight loss [29]. However, emerging evidence suggests that this approach may oversimplify the complex interplay of factors influencing energy balance and metabolic health.

Studies indicate that not all calories exert identical effects on metabolism. For instance, the thermic effect of food varies among macronutrients, with protein requiring more energy for digestion compared to carbohydrates and fats [30]. Additionally, individual differences in hormonal responses and genetic factors can influence how calories are processed and stored, challenging the notion of a one-size-fits-all approach to calorie counting [28,31].

Obesity is a chronic and multifactorial disease influenced not only by the quantity and quality of dietary intake, but also by the timing, frequency, and temporal distribution of meals. Accordingly, contemporary weight management strategies are increasingly incorporating these complex dimensions of eating behavior. In this context, the field of chrononutrition has emerged as a significant area of interest, emphasizing the role of meal timing and its alignment with endogenous circadian rhythms in the regulation of metabolic health and energy balance [32,33,34,35]. Circadian rhythms are internal, roughly 24 h cycles that control a range of physiological functions, such as metabolism, hormone release, and sleep–wake patterns [36,37,38]. Disruptions to these rhythms, such as irregular eating patterns or shift work, have been linked to adverse metabolic outcomes, including obesity and T2DM [39].

Research suggests that meal timing can significantly influence metabolic health. Consuming meals during the body’s active phase, typically earlier in the day, aligns with peak insulin sensitivity and glucose tolerance [40]. Conversely, late-night eating has been associated with impaired glucose metabolism and increased fat storage [41]. These findings highlight the potential of chrononutrition as a complementary approach to traditional dietary strategies [42].

This review aims to explore the interplay between meal timing, circadian rhythms, and energy balance, emphasizing their collective impact on weight regulation and metabolic health. The literature discussed in this narrative review was identified through a targeted search of PubMed and Scopus databases, focusing on observational, experimental, and review studies published between 2010 and 2025 related to the main topics of the review. Seminal papers published before this period were also included when relevant. We will examine the basic mechanisms by which circadian rhythms influence metabolic processes, the effects of meal timing on energy homeostasis, and the implications for lipid metabolism. Furthermore, we will discuss targeted nutritional interventions, such as time-restricted eating (TRE), that leverage chrononutrition principles to improve metabolic outcomes.

## 2. Foundations of Chronobiology and Circadian Rhythms

Circadian rhythms are internal cycles lasting about 24 h that regulate countless physiological and behavioral functions in living organisms, including sleep–wake cycles, hormone secretion, metabolism, and feeding behaviors [36]. These rhythms are governed by an intricate network of biological clocks that align internal processes with external environmental signals, chiefly the light–dark cycle. In mammals, the master clock is located in the suprachiasmatic nucleus (SCN) of the hypothalamus, which synchronizes peripheral clocks found in nearly all cells and tissues. These local clocks are now believed to independently regulate rhythmic activities specific to their respective tissues [43].

The SCN, situated above the optic chiasm, receives direct photic input from the retina via the retinohypothalamic tract. This input allows the SCN to align internal timekeeping with the external light–dark cycle, thereby regulating daily physiological rhythms [44]. The SCN exerts its influence through neural and hormonal signals, synchronizing peripheral clocks found in tissues such as the liver, pancreas, adipose tissue, and skeletal muscle. These peripheral clocks, while capable of maintaining autonomous rhythms, rely on cues from the SCN to remain in phase with the external environment. Disruption of this synchronization can lead to metabolic dysregulation and disease [43].

On a molecular level, circadian rhythms are driven by feedback loops of transcription and translation that involve key clock genes and the proteins they produce [36]. In mammals, the primary components include the transcription factors CLOCK and BMAL1, which heterodimerize to activate the expression of Period (Per1, Per2) and Cryptochrome (Cry1, Cry2) genes [45]. PER and CRY proteins build up in the cytoplasm, form complexes, and then move back into the nucleus, where they suppress their own gene expression by inhibiting CLOCK–BMAL1 activity. This negative feedback loop generates oscillations that follow a cycle of about 24 h [46,47,48]. Additional regulatory loops and post-translational modifications (including phosphorylation, acetylation, and ubiquitination) further refine the stability and precision of the circadian clock [49].

### 2.1. The Interplay Between Hormonal Modulation and Circadian Rhythms

Circadian regulation extends profoundly into the endocrine system, with several hormones displaying pronounced diurnal fluctuations. The rhythmic secretion of these hormones orchestrates systemic physiological states appropriate for different phases of the day–night cycle [50,51,52].

Melatonin, produced by the pineal gland, serves as a key marker of the circadian phase. Its synthesis is initiated in response to darkness and suppressed by light exposure, under direct control of the SCN via a multi-synaptic pathway. Melatonin promotes sleep initiation and exerts chronobiotic effects on peripheral tissues by resetting peripheral clocks via melatonin receptors expressed in tissues like liver and adipose, enhances the amplitude of clock gene expression (e.g., BMAL1) and synchronizes metabolic rhythmicity, reinforcing circadian synchronization [53,54].

Cortisol, the principal glucocorticoid in humans, follows a different circadian rhythm characterized by a sharp rise in the early morning hours and a gradual decline throughout the day. This diurnal variation prepares the body for the upcoming active period by promoting gluconeogenesis, immune modulation, and arousal [55,56,57].

Circadian rhythms also affect insulin secretion, with greater glucose tolerance and insulin sensitivity typically occurring during the early active phase. Disruptions to the circadian clock in pancreatic β-cells [58,59]—caused by factors like shift work, late-night eating, or genetic alterations—can impair insulin release and disrupt glucose regulation, leading to poor blood sugar control and a higher risk of developing type 2 diabetes [60,61].

Appetite-regulating hormones, such as ghrelin and leptin, are similarly regulated by circadian mechanisms. Ghrelin levels rise preprandially, promoting hunger, whereas leptin secretion peaks during the sleep phase, promoting satiety and reducing appetite [62,63]. Circadian misalignment alters the normal secretion patterns of these hormones, contributing to dysregulated appetite and weight gain [63,64,65].

Moreover, circadian disruption alters the expression of appetite-regulating neuropeptides in the hypothalamus, including neuropeptide Y (NPY), agouti-related peptide (AgRP), and pro-opiomelanocortin (POMC), further exacerbating imbalances between hunger and satiety signaling [62,66]. Night-shift workers, who experience chronic circadian misalignment, often exhibit increased caloric intake, preference for energy-dense foods, and impaired satiety responses, emphasizing the pivotal role of circadian timing in appetite regulation [55].

### 2.2. Synchronization of Biological Clocks: Entrainment Mechanisms

The process of aligning endogenous circadian rhythms with environmental cycles is known as entrainment [67]. Light is the principal zeitgeber (time-giver) for the SCN and the broader circadian system. Specialized photoreceptive retinal ganglion cells expressing melanopsin sense ambient light and transmit this information to the SCN, which adjusts its phase accordingly. This photic entrainment ensures that the endogenous clock remains in synchrony with the 24 h day [67,68,69].

However, feeding patterns emerge as potent zeitgebers for peripheral clocks [70]. Experimental studies in animals demonstrate that scheduled feeding can shift the phase of peripheral oscillators independently of the SCN, particularly in metabolic organs such as the liver and pancreas [71,72,73]. Some of the liver functions regulated by the circadian clocks include nutrient uptake, glucose and lipid metabolism, and amino acid processing, aligning them with feeding–fasting cycles. The liver clock can function independently of the central SCN clock, with feeding acting as a dominant time cue. Rhythmic gene expression is shaped by molecular clock components (e.g., CLOCK–BMAL1) and is influenced by intercellular communication and peripheral clock interactions. Recent studies also highlight spatial (zonation) and temporal coordination of liver metabolism, as well as emerging roles for ultradian rhythms [74]. This phenomenon suggests that while light primarily entrains the central clock, food intake timing predominantly entrains peripheral clocks [75,76,77,78].

The misalignment between light-driven and food-driven entrainment pathways, such as that seen in the central and peripheral clocks of night-shift workers who eat at biologically inappropriate times [55]. Chronodisruption of this nature has been related to the pathogenesis of obesity, insulin resistance, and cardiovascular diseases [40,79,80,81].

## 3. Mechanisms Linking Circadian Rhythms to Energy Metabolism

### 3.1. Regulation of Appetite and Satiety

Circadian rhythms exert a deep influence on the regulation of appetite and satiety through coordinated hormonal and neuronal signaling. The master clock within the SCN orchestrates daily fluctuations in the secretion of key orexigenic and anorexigenic hormones, notably ghrelin and leptin, respectively. Ghrelin, primarily secreted by the stomach, displays a preprandial rise, signaling hunger and promoting food intake. In contrast, leptin, predominantly produced by adipocytes, peaks during the nocturnal period to suppress appetite and promote energy storage [66,82,83].

Temporal misalignment between endogenous hormonal rhythms and actual eating behavior, such as consuming food during the biological night, disrupts this finely tuned balance, predisposing individuals to hyperphagia and weight gain [75,84,85]. Studies in rodents and humans have proven that food intake during the inactive/rest phase leads to greater weight gain compared to equivalent caloric intake during the active phase, independent of total energy consumed [41,86,87,88,89].

Moreover, circadian disruption alters the expression of appetite-regulating neuropeptides in the hypothalamus, including the amplification of neuropeptide Y (NPY), agouti-related peptide (AgRP) expression, and reduction of pro-opiomelanocortin (POMC) expression, further exacerbating imbalances between hunger and satiety signaling [62,80]. Night-shift workers, who experience chronic circadian misalignment, often exhibit increased caloric intake, preference for energy-dense foods, and impaired satiety responses, emphasizing the pivotal role of circadian timing in appetite regulation [55].

### 3.2. Impact on Insulin Sensitivity, Glucose Metabolism, and Lipid Storage

The circadian system tightly regulates glucose metabolism through rhythmic modulation of insulin sensitivity, pancreatic insulin secretion, and hepatic glucose production. Insulin sensitivity follows a diurnal pattern, peaking during the morning and declining toward the evening, coinciding with periods of greater metabolic efficiency. Despite high morning cortisol promoting gluconeogenesis, insulin sensitivity is also greatest in the morning. Cortisol aids glucose availability, while insulin receptors and downstream signaling in muscle/liver are most responsive earlier in the day due to robust peripheral clock gene expression [60,90,91,92].

A cross over study demonstrates that eating at biological night—when insulin sensitivity is reduced—leads to higher postprandial glucose excursions and impaired glucose tolerance, independent of meal composition or quantity [93] and results of a systematic review and meta-analysis of acute postprandial studies suggest poor glucose tolerance at night compared to the day [94]. Similarly, the timing of meals relative to circadian phase significantly influences glycemic responses; late-night eating is consistently associated with elevated glucose levels and increased risk for T2DM [39,95].

Molecular clock components within pancreatic β-cells, such as CLOCK and BMAL1, regulate the timing and amplitude of insulin secretion. Genetic disruption of these core clock genes impairs glucose-stimulated insulin secretion and predisposes to diabetes [96]. Furthermore, hepatic glucose production is subject to circadian regulation, with clock genes modulating the expression of key gluconeogenic enzymes such as phosphoenolpyruvate carboxykinase (PEPCK) and glucose-6-phosphatase (G6Pase). The CLOCK–BMAL1 heterodimer binds to E-box elements in promoters of Pck1 (PEPCK), G6pc, and other gluconeogenic genes, activating transcription in a time-of-day-specific fashion. Additionally, CRY proteins suppress CREB-mediated transcription of gluconeogenic genes during fasting [97,98].

Beyond glucose homeostasis, circadian rhythms play a crucial role in lipid metabolism, including the regulation of lipogenesis, lipolysis, and lipid transport. There are key genes involved in lipid metabolism, such as sterol regulatory element-binding protein-1c (SREBP-1c), as it peaks during feeding times, promoting lipogenesis during the day; peroxisome proliferator-activated receptor gamma (PPARγ), which oscillates with circadian control via BMAL1 and PER2, regulating adipogenesis; and acetyl-CoA carboxylase (ACC), the activity of which activity follows feeding rhythms, with higher expression during the day promoting fatty acid synthesis. The mentioned genes exhibit circadian expression patterns that align lipid biosynthesis with periods of feeding and fasting [99,100,101].

Adipose tissue clocks modulate lipolytic activity in a time-dependent manner, promoting lipid mobilization during fasting phases and lipid storage during feeding [102,103]. Disruption of circadian rhythms, whether through genetic manipulation or behavioral perturbations, leads to dysregulated lipid metabolism characterized by increased triglyceride accumulation, ectopic fat deposition, and elevated plasma free fatty acids [103,104].

Importantly, the timing of food intake impacts lipid profiles. Animal studies reveal that high-fat meals consumed during the rest phase exacerbate lipid accumulation and impair metabolic flexibility compared to equivalent meals consumed during the active phase (as in the time-restricted dietary strategy) [105]. Human observational studies corroborate these findings, showing that late-night eating patterns are associated with dyslipidemia, increased visceral adiposity, and heightened risk of metabolic syndrome [106,107].

### 3.3. Thermogenesis and Diurnal Variations in Energy Expenditure

Energy expenditure exhibits marked circadian variation, reflecting the temporal organization of metabolic processes. Components of total daily energy expenditure, including basal metabolic rate (BMR), diet-induced thermogenesis (DIT), and activity energy expenditure (AEE), are influenced by circadian rhythms [108,109,110].

Resting energy expenditure is generally higher during the biological morning and early afternoon compared to the evening and night [111]. Controlled studies employing constant routine protocols confirm that endogenous circadian rhythms contribute to fluctuations in metabolic rate independent of behavioral factors [111,112,113].

Diet-induced thermogenesis—the energy the body uses for digesting, absorbing, and storing nutrients from food—also follows a circadian pattern, being greater in response to morning meals compared to evening meals. This suggests that consuming larger meals earlier in the day may confer metabolic advantages by enhancing energy expenditure and reducing postprandial glucose and lipid excursions [41,114,115,116].

Brown adipose tissue (BAT)-mediated thermogenesis, a process crucial for non-shivering heat production, is under circadian control as well. The expression of thermogenic genes such as uncoupling protein 1 (UCP1) in BAT peaks during the active phase, aligning heat production with periods of increased energy demand [117,118,119].

**Figure 1:**
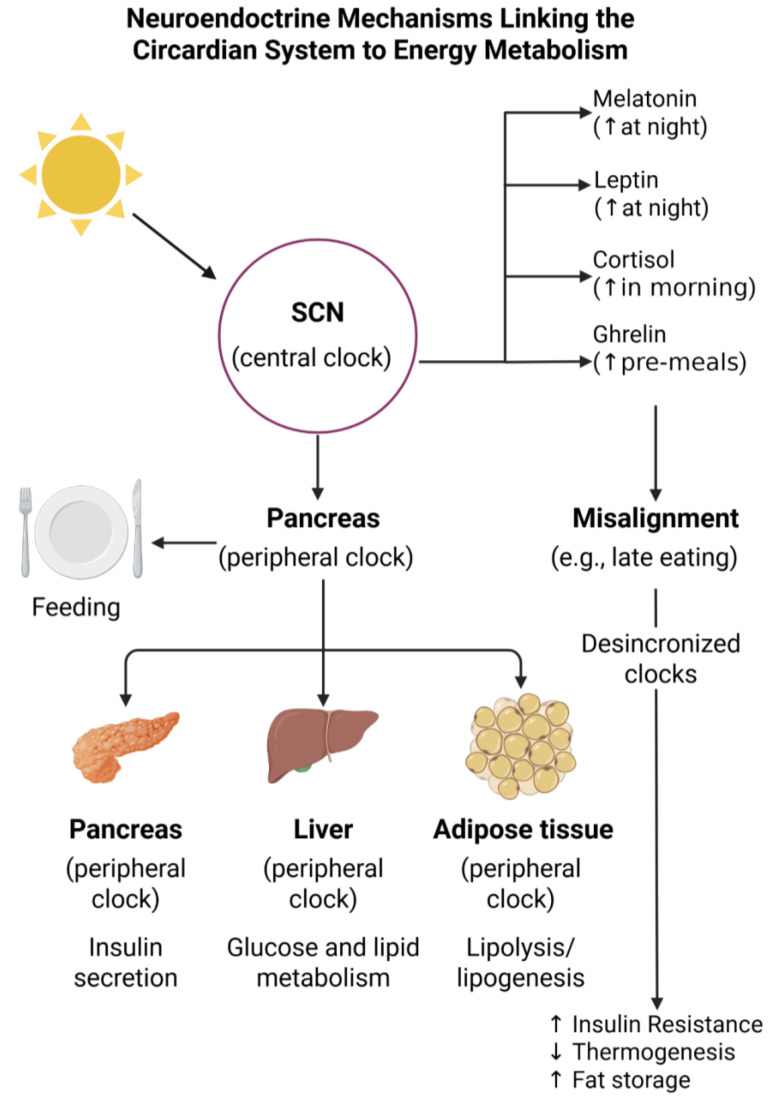
Illustration of the bidirectional relationship between the circadian system and metabolic regulation. It emphasizes how feeding behavior and light exposure interact with the master clock in the SCN and peripheral clocks in metabolic tissues. Disruption of this network, particularly through inappropriate meal timing, contributes to metabolic dysregulation, including impaired insulin sensitivity, altered lipid metabolism, and increased risk for obesity [62,63]. Abbreviations: SCN = suprachiasmatic nucleus.

Circadian disruption blunts these rhythms in energy expenditure, contributing to positive energy balance and weight gain over time (Figure 1). Consequently, chrononutrition strategies aiming to align food intake with periods of higher metabolic efficiency offer promising avenues for obesity prevention and treatment [33].

### 3.4. Hypothalamic Integration of Peripheral and Central Signals

The hypothalamus functions as a key center for integrating central circadian signals with peripheral metabolic inputs. Within this region, various nuclei—such as the arcuate nucleus (ARC), ventromedial hypothalamus (VMH), dorsomedial hypothalamus (DMH), and lateral hypothalamus (LH)—work together through intricate neuroendocrine pathways to regulate energy balance [66,120,121].

The ARC contains two primary populations of neurons: the orexigenic NPY/AgRP neurons and the anorexigenic POMC/CART neurons. These neurons process hormonal signals—including leptin, insulin, ghrelin, and peptide YY (PYY)—to regulate feeding behavior and energy expenditure in response to the body’s metabolic needs [122,123,124].

Circadian input from the SCN to the hypothalamus modulates the rhythmicity of these metabolic processes. For instance, the SCN projects to the paraventricular nucleus (PVN), influencing the secretion of corticotropin-releasing hormone (CRH) and subsequently regulating glucocorticoid rhythms, which are essential for maintaining energy homeostasis [125,126,127].

Moreover, feeding-related cues provide feedback to the central clock, creating a bidirectional relationship between metabolism and circadian timing. Peripheral metabolic signals, including circulating levels of glucose, free fatty acids, and ketone bodies, can influence hypothalamic activity and, in turn, modulate behavioral outputs such as hunger, satiety, and thermogenesis [128,129,130].

Evidence suggests that disruption of hypothalamic clock genes impairs the ability to appropriately respond to metabolic signals, leading to hyperphagia, reduced energy expenditure, and obesity. For example, selective deletion of Bmal1 in the VMH results in impaired leptin sensitivity and dysregulated body weight control [131,132].

Thus, the hypothalamus operates as an integrative center where circadian information and metabolic status converge to regulate energy balance dynamically. Interventions that reinforce circadian synchrony within hypothalamic circuits may offer novel therapeutic approaches for combating obesity and metabolic disease.

## 4. Meal Timing and Metabolic Outcomes

### 4.1. Early Versus Late Eating Patterns: Insights from Human and Animal Studies

Food intake timing plays a crucial role in metabolic health. Numerous studies have demonstrated that early eating patterns, in which the majority of caloric intake occurs earlier in the day, are associated with improved metabolic outcomes compared to late eating patterns.

In human studies, early time-restricted eating (eTRE)—consuming meals within the early part of the day—has shown beneficial effects on weight regulation, insulin sensitivity, and lipid profiles [133]. A randomized controlled trial conducted in 2025 by Yu et al. in young adult women demonstrated that eTRE, with food intake confined to an 8:00 AM–2:00 PM window, led to significant reductions in body weight while preserving lean muscle mass. These outcomes were superior when compared to both a delayed TRE group (12:00 PM–6:00 PM) and a control group following a conventional eating window (8:00 AM–8:00 PM) [134]. Similarly, a 2022 randomized clinical trial involving 90 adults with obesity compared the effects of eTRE combined with energy restriction (an 8 h eating window from 7:00 AM to 3:00 PM) versus energy restriction alone with a ≥12 h eating window. The findings revealed that eTRE was more effective in promoting weight loss, improving diastolic blood pressure, and enhancing mood relative to the control condition [135].

However, there is ongoing debate on whether early or late is the optimal timing of the eating window in TRE intervention. To address this, a recent network meta-analysis evaluated the comparative efficacy of early versus late TRE on weight loss and metabolic health outcomes in adults who were overweight or obese. The analysis included twelve randomized controlled trials encompassing a total of 730 participants, with data sourced from PubMed, Embase, Web of Science, and the Cochrane Library up to 16 October 2022. Both early and late TRE protocols were found to induce moderate reductions in body weight and insulin resistance, as assessed by the homeostasis model assessment of insulin resistance (HOMA-IR), relative to non-TRE controls. Notably, eTRE was significantly more effective than late TRE in improving insulin resistance (mean difference: −0.44; 95% CI, −0.86 to −0.02; *p* < 0.05), although no significant difference was observed between the two interventions in terms of weight loss (mean difference: −0.31 kg; 95% CI, −1.15 to 0.53 kg; *p* > 0.05). Furthermore, eTRE showed superior benefits in glycemic control and blood pressure regulation compared to non-TRE, whereas these advantages were not statistically significant for late TRE. However, no meaningful differences were detected between early and late TRE regarding fasting glucose levels, blood pressure, or lipid profiles. These findings suggest a potential metabolic advantage of early TRE, though larger and more robust clinical trials are needed to substantiate these preliminary observations [133].

Similarly, animal studies have supported these observations. Mice that underwent time-restricted feeding during their active phase (comparable to daytime in humans) showed a reset in clock genes and genes related to lipid metabolism, mediated by nutrient-sensing pathways in the liver, brown fat, and peri-epididymal adipose tissue. In contrast, mice fed during their inactive phase or given unrestricted access to food did not exhibit these changes [71].

Another study in animals supports this information. In the study by de Goede et al., 45 male Wistar rats were assigned to three feeding conditions—ad libitum (AL), TRE during the light phase (light-TRE), or dark phase (dark-TRE)—for four weeks to assess how feeding time relative to circadian phase affects glucose metabolism. Using intravenous glucose tolerance tests (ivGTT), the study found that TRE aligned with the active (dark) phase improved insulin sensitivity, as evidenced by reduced insulin levels with similar glucose clearance compared to other groups. In contrast, TRE during the inactive (light) phase did not improve glucose tolerance, likely due to chronic adaptation and prolonged fasting benefits. The study demonstrated that both circadian timing and feeding-fasting patterns influenced metabolic outcomes, with the most favorable glucose handling observed when TRE was synchronized with the natural active phase. These findings suggest that adherence to circadian-aligned feeding windows may enhance the effectiveness of TRE in metabolic regulation [136].

More updated studies in animals show similar insights of TRE patterns. In the 2024 study by Yang and Liu, middle-aged and old high-fat-diet-induced obese male mice underwent an 8 h daily TRE regimen for 8 weeks to assess its effects on obesity and metabolic health in aged subjects. Clinically relevant findings include that while TRE did not reduce overall fat mass in aged mice, it led to a loss of lean mass—raising concerns given the importance of muscle preservation in aging. Despite this, TRE significantly improved metabolic parameters by reducing brown adipose tissue adiposity, reversing hepatic lipid accumulation, and enhancing glucose homeostasis. These effects were attributed to downregulation of genes involved in hepatic adipogenesis and adipose tissue inflammation. The study emphasizes the potential of TRE to restore metabolic health in aged obese mice, but also underscores the need for complementary strategies to preserve lean mass in older populations undergoing dietary interventions [137].

Overall, both preclinical and clinical data underline that aligning eating patterns with the body’s natural circadian rhythm—favoring early-day energy intake—may optimize metabolic outcomes.

### 4.2. Effects of Breakfast Skipping, Late-Night Eating, and TRE Impact on Weight Regulation, Fat Distribution, and Metabolic Syndrome Risk Factors

Breakfast is currently considered the most important meal of the day for metabolic regulation [138,139]. Emerging evidence continues to support this notion. Skipping breakfast has been linked to increased risk of obesity, T2DM, and cardiovascular diseases [140,141,142].

A 2019 systematic review and meta-analysis of prospective cohort studies found an association between skipping breakfast and an increased risk of developing T2DM, with the risk rising by 33% compared to those who regularly eat breakfast. Even after adjusting for body mass index (BMI), the increased risk remained significant at 22%, indicating that adiposity only partially explains the association. Based on data from six cohort studies involving 96,175 participants and 4935 diabetes cases, the analysis revealed a nonlinear dose–response relationship: the risk of T2DM increased with each additional day of breakfast skipping but plateaued after 4–5 days per week, showing a maximum relative risk of 1.55. These findings suggest that frequent breakfast skipping is an independent and modifiable risk factor for T2DM [143].

Building on the evidence that breakfast skipping increases the risk of T2DM, a 2019 systematic review of four large prospective cohort studies involving 199,634 adults further indicates that skipping breakfast is also associated with a significantly higher risk of cardiovascular events and mortality. Over a median follow-up of 17.4 years, individuals who regularly skipped breakfast were found to have a 21% increased risk of developing or dying from cardiovascular disease and a 32% higher risk of all-cause mortality compared to those who ate breakfast consistently. Although these findings are compelling, the studies varied in how they defined breakfast skipping and adjusted for confounding variables, suggesting that while the association is strong, causality cannot be firmly established without further large-scale, standardized research. Together, these results support the clinical recommendation of regular breakfast consumption as a potentially important strategy for cardiometabolic disease prevention [144].

Late-night eating has emerged as a detrimental behavior for metabolic health. Consuming meals close to the biological night—when endogenous melatonin levels rise—impairs glucose tolerance and lipid metabolism [52].

A 2022 randomized, controlled, crossover trial by Vujović et al. found that late eating, compared to early eating, significantly increased hunger (*p* < 0.0001) and disrupted appetite-regulating hormones, notably raising the ghrelin-to-leptin ratio (*p* < 0.0001 and *p* = 0.006, respectively). It also reduced waketime energy expenditure (*p* = 0.002) and core body temperature (*p* = 0.019), suggesting a lower metabolic rate. Transcriptomic analysis of adipose tissue revealed that late eating altered key metabolic pathways—such as p38 MAPK and TGF-β signaling—toward decreased lipolysis and increased adipogenesis. These findings suggest that late eating may promote a positive energy balance and increase the risk of obesity through hormonal, metabolic, and gene expression changes [145].

Additionally, late-night eating is associated with desynchronization between central and peripheral clocks. Nighttime ingestion leads to inappropriate activation of nutrient-sensing pathways (e.g., AMPK, mTOR) at a time when the body is primed for fasting and cellular repair, promoting metabolic dysfunction [146,147,148].

Regarding time-restricted eating, a systematic review and meta-analysis by Fernandes-Alves et al., evaluated the effects of TRE, with and without caloric restriction (CR), on body weight and composition in adults with overweight or obesity. Analyzing 30 randomized controlled trials comprising 1341 participants, the study found that TRE significantly reduced body weight, fat mass, and fat-free mass in both non-isocaloric (mean difference [MD] in body weight: −2.82 kg; 95% CI: −3.49, −2.15; fat mass: −1.36 kg; 95% CI: −2.09, −0.63; fat-free mass: −0.86 kg; 95% CI: −1.23, −0.49) and isocaloric conditions (body weight: −1.46 kg; 95% CI: −2.65, −0.26; fat mass: −1.50 kg; 95% CI: −2.77, −0.24; fat-free mass: −0.41 kg; 95% CI: −0.79, −0.03). These results suggest that TRE offers beneficial anthropometric outcomes even when caloric intake is controlled, indicating that circadian mechanisms may augment the metabolic effects of CR [149].

Several reviews on this topic have demonstrated that TRE exhibits a range of beneficial metabolic effects, some of which occur independently of CR. Evidence from multiple randomized trials indicates that TRE can reduce body weight, fat mass, body mass index (BMI), waist circumference, fasting glucose, insulin resistance, systolic blood pressure, triglycerides, and total cholesterol levels. Specifically, eTRE in isocaloric conditions has improved glycemic control and insulin sensitivity and even achieve blood pressure reductions comparable to pharmacological interventions. Notably, TRE has been associated with increases in adiponectin, a fasting-induced adipokine that enhances insulin sensitivity and stimulates lipolysis via AMPK activation, potentially explaining fat mass reduction independent of caloric intake. Some studies also report increased HDL cholesterol levels [150,151,152,153]. Table 1 shows a comparison of the key metabolic outcomes in the additional cited systematic reviews. However, the role of physical activity in mediating these effects remains underexplored, as only a few trials have systematically measured or controlled for physical activity levels. Thus, avoiding late-night meals and adopting early or mid-day time-restricted feeding appears to optimize metabolic outcomes.

This table compares findings from four systematic reviews investigating the metabolic effects of TRE in various contexts. The study by Réda et al. (2020) [150] highlights the metabolic health benefits of TRE even without CR, attributing them to circadian rhythm alignment. Kamarul Zaman et al. (2023) [151] report that an 8 h eating window is more effective for weight loss than longer windows, particularly in interventions exceeding 12 weeks. Silva et al. (2025) [152] note an increase in hunger among overweight or obese adults undergoing TRE, which may impact adherence. Finally, Hays et al. (2025) [153] demonstrate that combining TRE with physical exercise can reduce fat mass without compromising muscle mass. Collectively, these studies suggest that TRE can be an effective strategy to improve metabolic health, especially when tailored to individual needs and combined with exercise.

### 4.3. Macronutrient Timing

Emerging research indicates that not only when we eat, but also which macronutrients are consumed at specific times of the day, significantly influences metabolic health [41].

### 4.4. Carbohydrate Timing

A 2021 randomized, open-label, parallel-group study that included 43 patients diagnosed with T2DM investigated how the timing of carbohydrate intake—restricted to either breakfast or dinner—impacts glucose fluctuations and energy metabolism [154]. Both groups followed an isocaloric, carbohydrate-restricted diet (10% carbohydrate at one meal only) over two days. The intervention led to a significant reduction in both average 24 h blood glucose levels and postprandial glucose spikes in both groups. Notably, carbohydrate restriction at breakfast led to a significant increase in glucose levels after lunch (iAUC_0–2_h), a reduction in diet-induced thermogenesis at breakfast (223 ± 117 to 109 ± 104 kcal, *p* = 0.002), and a marked rise in pre-lunch plasma free fatty acids (0.20 ± 0.09 to 0.63 ± 0.19 mEq/L, *p* < 0.001), effects not observed with dinner-time restriction. These findings suggest that the timing of carbohydrate intake modulates glycemic control and metabolic responses in a meal-specific manner in patients with T2DM.

In a previous analysis of 6155 UK adults from the National Diet and Nutrition Survey (2008/09–2015/16), Wang et al. (2019) identified day-time carbohydrate (CH) intake patterns and examine their association with T2DM [155]. Three distinct CH eating profiles emerged: low-CH eaters (28.1%), moderate-CH eaters (28.8%), and high-CH eaters (43.1%). High-CH eaters consumed most of their CH during traditional meal times (6–9 a.m., 12–2 p.m., 5–8 p.m.) and had the highest fiber and lowest fat and protein intake. In contrast, low-CH eaters consumed more total daily energy—especially from fat and alcohol—primarily in the evening (after 8 p.m.) and had significantly higher odds of self-reported T2D compared to high-CH eaters. These findings suggest that not only the quantity but also the timing of carbohydrate intake may influence metabolic health, highlighting the potential role of chrononutrition in T2D risk.

### 4.5. Protein Timing

A multicenter, double-blind randomized controlled study from the Full4Health project by Crabtree et al., investigated the acute effects of breakfast drink quantity and protein content on appetite control across age groups, weight categories, genders, and European sites (Scotland and Greece) in 391 participants. Subjects consumed four test drinks varying in protein content (15% vs. 30% of energy) and volume (100% vs. 140% of basal metabolic rate) across four separate days. While subjective appetite was significantly lower in elderly participants compared to adults (*p* < 0.004), no differences were observed in ad libitum energy intake across age, weight, gender, or site. Notably, high-protein drinks significantly elevated postprandial concentrations of anorectic hormones GLP-1 and PYY (*p* < 0.001), particularly in older adults. Additionally, GLP-1 and PYY levels were higher in the elderly, while ghrelin and fasting leptin levels varied significantly across weight categories, genders, and sites (*p* < 0.05) [156]. This study focused on protein quantity and age differences, offering insight into how breakfast composition and volume (nutrient timing) may differentially affect appetite hormones (GLP-1, PYY, and ghrelin) depending on the time of day they are consumed.

A systematic review and meta-analysis conducted by Nunes et al. (2022), which analyzed 74 randomized controlled trials, assessed the effects of increased daily protein intake on lean body mass (LBM), muscle strength, and physical function in healthy, non-obese adults [157]. The findings show that higher protein intake, particularly when combined with resistance exercise (RE), led to modest but significant gains in LBM (SMD = 0.22; 95% CI: 0.14–0.30; *p* < 0.01). These effects were most pronounced in older adults (≥65 years) consuming 1.2–1.59 g/kg/day and in younger adults (<65 years) consuming ≥1.6 g/kg/day during RE. Additional protein intake also slightly improved lower-body strength (SMD = 0.40) and bench press strength in younger adults (SMD = 0.18), though effects on handgrip strength and overall physical function were minimal or unclear. Overall, the evidence supports increased protein intake as a beneficial strategy for enhancing muscle mass and lower-body strength when combined with RE, particularly in age- and dose-specific contexts.

Another systematic review and meta-analysis, by Wirth et al., encompassing 65 randomized controlled trials with 2907 healthy adults, evaluated the effects of protein supplementation on body composition and muscle function, with particular attention to intake timing. The findings demonstrate that protein supplementation significantly increased lean body mass (LBM) in both adults (mean difference: 0.62 kg; 95% CI: 0.36, 0.88) and older adults (0.46 kg; 95% CI: 0.23, 0.70), regardless of the timing of intake. However, improvements in muscle strength (handgrip and leg press) were not statistically significant, and evidence for enhanced muscle protein synthesis was limited and inconclusive. Importantly, subgroup and sensitivity analyses confirmed that the benefits to LBM were consistent even without concurrent exercise training, and that timing of protein intake had no additional effect on outcomes [158]. Although these findings suggest that total protein intake improves lean body mass regardless of timing, these findings must be interpreted within the context of emerging chrononutritional evidence. For instance, morning protein intake may align better with peak muscle protein synthesis and hormonal profiles, such as elevated insulin sensitivity and anabolic hormone levels earlier in the day.

## 5. TRE and Intermittent Fasting

Time-restricted eating (TRE) and intermittent fasting (IF) are dietary approaches that emphasize the timing of meals rather than the amount or type of food consumed. TRE involves confining daily food consumption to a specific time window, typically ranging from 4 to 10 h, followed by a fasting period of 14 to 20 h [159]. This approach aligns eating patterns with the body’s circadian rhythms, potentially enhancing metabolic health [153,159,160]. In contrast, IF encompasses various patterns, including alternate-day fasting, the 5:2 diet (five days of normal eating and two non-consecutive days of reduced caloric intake), and periodic fasting, where fasting occurs for extended periods intermittently [161,162,163].

These dietary patterns have gained popularity due to their simplicity and potential health benefits. Unlike traditional calorie-restricted diets, TRE and IF do not necessarily require calorie counting, making them more accessible to the general population [164,165,166,167]. The flexibility in choosing eating windows allows individuals to tailor these approaches to their lifestyles, potentially improving their adherence.

### 5.1. Evidence from Clinical Trials

Numerous clinical trials have explored the efficacy of TRE and IF in improving metabolic health outcomes. In a systematic review and meta-analysis by Dai et al., the authors evaluated the combined effects of TRE and exercise on body composition and metabolic health in adults, synthesizing data from 19 randomized controlled trials involving 568 participants. The findings demonstrate that TRE combined with exercise significantly reduced body mass (mean difference [MD]: −1.86 kg; 95% CI: −2.75 to −0.97 kg) and fat mass (MD: −1.52 kg; 95% CI: −2.07 to −0.97 kg) compared to a control diet paired with exercise. Additionally, this intervention lowered triglycerides (MD: −13.38 mg/dL), low-density lipoprotein cholesterol (LDL-C) (MD: −8.52 mg/dL), and leptin levels (MD: −0.67 ng/mL), indicating potential improvements in lipid metabolism and adiposity regulation. However, there were no notable differences in fasting glucose, insulin, total cholesterol, or HDL cholesterol levels. These results suggest that TRE, when combined with exercise, may be an effective strategy for improving body composition and select metabolic parameters in adults, although further high-quality trials are needed to confirm its long-term efficacy and wider metabolic impacts [168].

Previously, in a 2023 umbrella review—a high-level synthesis that evaluates and integrates findings from multiple systematic reviews and meta-analyses—Chew et al. assessed the efficacy of TRE on weight loss, fasting blood glucose, and lipid profiles in individuals who were overweight and obese, synthesizing evidence from 7 systematic reviews and 30 meta-analyses involving 7231 participants across 184 primary studies. The analysis reveals that TRE significantly reduced body weight (*p* = 0.006), fasting blood glucose (*p* < 0.01), and low-density lipoprotein cholesterol (LDL-C) (*p* = 0.03). Subgroup analyses indicated that Ramadan fasting was more effective than conventional TRE in lowering fasting glucose, total cholesterol, and LDL-C. However, the credibility of evidence was limited, with only 14.3% of reviews rated as moderate quality and 85.7% as critically low, and just 3.3% of associations classified as suggestive. These findings suggest that while TRE shows potential as a dietary strategy for improving cardiometabolic health, higher-quality randomized controlled trials with diverse populations and extended follow-up durations are necessary to confirm its clinical utility [169].

Regarding IF, an umbrella review by Sun et al. analyzed 23 meta-analyses encompassing 351 associations to assess the effects of IF on health outcomes. The review, based on randomized controlled trials and rated with high methodological quality (91% high confidence per AMSTAR), found that IF significantly improved multiple cardiometabolic parameters in adults with obesity or who were overweight. Specifically, IF reduced waist circumference (MD = –1.02 cm), fat mass (MD = –0.72 kg), fasting insulin (SMD = –0.21), LDL cholesterol (SMD = –0.20), total cholesterol (SMD = –0.29), and triacylglycerols (SMD = –0.23), while increasing fat-free mass (MD = +0.98 kg) and HDL cholesterol (MD = +0.03 mmol/L). Notably, although IF showed broad benefits compared to non-intervention diets and sometimes continuous energy restriction (CER), its systolic blood pressure-lowering effect was less pronounced than that of CER. These findings suggest IF as a promising dietary intervention to improve metabolic health, though evidence quality varies across outcomes and warrants further targeted investigation [170]. These findings complement and reinforce the conclusions previously drawn by Patikorn et al. in a similar umbrella review [171].

### 5.2. Mechanistic Insights: Autophagy, Insulin Sensitivity, and Mitochondrial Function

One of the foundational mechanisms through which TRE and IF confer metabolic benefits is the stimulation of autophagy. During periods of fasting, the reduction in nutrient and insulin signaling activates autophagy, a highly conserved cellular process that involves the degradation and recycling of damaged or dysfunctional organelles, misfolded proteins, and other cellular debris. This self-cleaning mechanism enhances cellular function and resilience, contributing to improved metabolic efficiency. By clearing intracellular clutter and supporting cellular renewal, autophagy helps to prevent the accumulation of oxidative damage, inflammation, and mitochondrial dysfunction—all of which are key drivers of metabolic diseases such as obesity, insulin resistance, and T2DM [172,173,174,175,176]. Furthermore, enhanced autophagy has been linked to protection against age-related metabolic decline and chronic conditions like nonalcoholic fatty liver disease and cardiovascular disease [6,100].

Another major mechanism underpinning the metabolic effects of TRE and IF is the improvement in insulin sensitivity. Fasting periods naturally lower circulating insulin levels and allow insulin receptors to regain sensitivity, particularly in peripheral tissues such as muscle and adipose tissue. This effect reduces the burden on pancreatic beta cells and improves glucose uptake and utilization, ultimately leading to better glycemic control. Clinical and experimental studies have demonstrated that regular fasting cycles can decrease fasting insulin levels, reduce insulin resistance markers, and enhance the insulin-mediated glucose disposal rate. These changes are especially beneficial for individuals with metabolic syndrome or T2DM, as they help restore glucose homeostasis and lower the risk of disease progression [172,177]. Additionally, aligning food intake with circadian rhythms appears to amplify these insulin-sensitizing effects, suggesting that meal timing is just as critical as fasting duration.

Fasting also promotes improved mitochondrial health, another critical factor in metabolic regulation. TRE and IF can trigger mitochondrial biogenesis—the process by which new mitochondria are formed—and enhance mitochondrial efficiency, leading to more effective ATP production and reduced reactive oxygen species (ROS) generation. These adaptations result in better energy utilization and reduced metabolic stress, contributing to improved endurance, fat oxidation, and overall metabolic flexibility. Enhanced mitochondrial function not only supports the energetic demands of cells but also plays a protective role against metabolic diseases by decreasing oxidative damage and preserving cellular homeostasis. Studies in both animal models and humans suggest that fasting-induced improvements in mitochondrial quality control are associated with delayed aging, reduced inflammation, and lower risk of obesity-related complications [178,179]. Thus, the enhancement of mitochondrial dynamics represents a key avenue through which TRE and IF exert systemic metabolic benefits [180].

### 5.3. Benefits and Challenges in Long-Term Adherence

While TRE and IF offer promising health benefits, their long-term adherence poses challenges. Factors influencing adherence include individual lifestyle, work schedules, social commitments, and personal preferences.

TRE appears to offer meaningful cardiometabolic benefits, particularly in supporting weight management and improving metabolic markers, yet its real-world effectiveness is limited by variable adherence. Across studies, adherence rates to TRE protocols ranged widely from 47% to 95% of days, highlighting a significant challenge in consistent implementation. Reported barriers to adherence included family responsibilities, social events, work schedules, and other lifestyle factors, all of which can disrupt the eating window. These findings emphasize that while TRE may be physiologically beneficial, its success in clinical and everyday settings depends heavily on behavioral and environmental factors. To enhance outcomes, future applications of TRE should incorporate structured behavioral interventions and personalized support strategies that address these common barriers, thereby improving adherence and the overall effectiveness of the dietary approach [181].

IF also offers numerous health benefits, including improved insulin sensitivity, enhanced body composition when paired with resistance training, and potential reductions in inflammation and emotional stress. These effects contribute to IF’s growing appeal as a strategy for managing metabolic health and weight. However, sustaining IF in the long term presents notable challenges. Adherence can be hindered by the restrictive nature of fasting protocols such as 16/8, 5:2, or alternate-day fasting, which may be difficult to integrate into daily routines, especially without individualized support. Despite its benefits, the success of IF depends heavily on a participant’s ability to maintain the regimen over time, which is influenced by lifestyle compatibility, social factors, and psychological resilience. Therefore, long-term effectiveness requires careful consideration of personal and behavioral factors, and the guidance of healthcare professionals to ensure sustainable adherence and minimize adverse effects [182].

Moreover, potential side effects such as hunger, irritability, and decreased energy levels during fasting periods may deter individuals from sustaining these dietary patterns. Therefore, healthcare professionals should consider individual circumstances when recommending TRE or IF and provide guidance to mitigate potential challenges.

## 6. Chrononutrition in Special Populations

In special populations, such as shift workers, adolescents, the elderly, or when considering sex differences, meal timing often deviates from the natural light–dark cycle. These deviations amplify disruptions of clock-controlled hormones (e.g., cortisol, melatonin, ghrelin, leptin) and core clock genes (CLOCK, BMAL1, PER, etc.), contributing to adverse metabolic outcomes.

### 6.1. Shift Workers and Metabolic Disruption

Shift work induces circadian misalignment by forcing wake–sleep and eating–fasting cycles to occur at “wrong” biological times. Night and rotating shift workers have substantially higher rates of obesity, metabolic syndrome, diabetes, and cardiovascular disease than day workers [146,183]. The misalignment originates in desynchrony of the central clock (SCN) and peripheral clocks: light exposure at night suppresses melatonin, while feeding at night (when the body is programmed for sleep) disrupts metabolic rhythms. For example, night-shift nurses exhibit blunted nocturnal melatonin secretion and altered cortisol rhythms [183,184]. In an observational study, night workers had attenuated cortisol secretion during work hours and more pronounced social jet lag than day workers [184], indicating HPA-axis dysregulation.

Metabolically, eating out of phase worsens glucose and lipid tolerance. In humans, late meals reduce postprandial energy expenditure and elevate glycemia. For instance, shifting a standard lunch from 13:00 to 16:30 lowered resting energy expenditure and worsened glucose handling [185].

At the molecular level, chronic shift work dampens clock gene expression. In rodent models of simulated shift schedules, CLOCK–BMAL1 binding in pancreatic β-cells weakens, reducing rhythmic gene transcription essential for insulin release [183].

### 6.2. Adolescents and Meal-Timing Irregularities

Adolescence is characterized by a late-shifted circadian phase and erratic eating schedules. Puberty delays endogenous melatonin release by 1–3 h, causing teens to feel sleepy later and often rise early for school [186,187]. As a result, many adolescents skip breakfast and consume a disproportionate amount of calories in the evening or at night.

A 2021 systematic review by Souza et al., encompassing 43 observational studies with a total of 192,262 adolescents aged 10 to 19 years, found that breakfast skipping is significantly associated with increased cardiometabolic risk factors, particularly excess adiposity, elevated levels of total cholesterol, LDL cholesterol, triglycerides, and high blood pressure. Despite these associations being observed in both cross-sectional and longitudinal analyses, no significant relationship was found between breakfast omission and glycemic control.

The prevalence of breakfast skipping ranged significantly among studies, from 0.7% to 94%. Although most studies showed a low risk of bias, the overall quality of the evidence was assessed as low according to the GRADE system. These findings suggest a potential link between skipping breakfast and poorer cardiometabolic health in adolescents, though the evidence remains inconclusive and underscores the need for standardized definitions and well-designed prospective studies to clarify long-term health implications [188].

### 6.3. Elderly Individuals and Altered Circadian Rhythms

Normal aging entails progressive weakening and phase-shifting of circadian systems [189,190]. Older adults tend to awaken and eat earlier (“morning chronotype”), experience fragmented sleep, and nap more. These behavioral changes reflect central clock deterioration: the SCN’s neuronal output (its firing amplitude) declines with age, and responsiveness to light diminishes. Consequently, rhythms of key hormones show advance and dampening. In elderly subjects, the nocturnal melatonin and cortisol peaks occur earlier and with reduced amplitude. Core body temperature rhythms likewise advance and flatten, contributing to earlier sleepiness and morning fatigue [191].

A scoping review by Ferreira et al. analyzed 42 studies investigating age-related differences in circadian rhythms of body fluid composition, highlighting significant disruptions in circadian regulation among the elderly. Across 48 identified parameters—including cortisol, melatonin, sex hormones, thyroid hormones, steroids, and aldosterone—most studies indicated a general flattening of circadian oscillations in older adults, potentially contributing to age-related physiological decline and disease. However, findings were inconsistent across studies, with some parameters showing no age-related changes or yielding contradictory results. Despite the volume of research, the clinical implications of altered circadian rhythms in aging remain unclear, emphasizing the need for further targeted studies to clarify their impact on health outcomes in the elderly population [192].

### 6.4. Gender-Specific Considerations

Sex differences permeate circadian physiology and chrononutrition outcomes [193]. Men and women differ in clock gene regulation, hormone profiles, and body composition—factors that modify how meal timing affects metabolism. For example, females generally have higher 24 h leptin levels (even when BMI-matched) and a slightly shorter intrinsic circadian period than males. These differences mean that identical eating schedules may yield divergent hormonal responses by sex [194].

Experimental evidence highlights this dimorphism. In lab protocols of circadian misalignment, women suffered greater metabolic derangement than men. Specifically, Scheer’s group showed that under shifted sleep–feeding cycles, women exhibited decreased leptin and increased active ghrelin, whereas men had the opposite (increased leptin, stable ghrelin). This suggests that misalignment elicits a stronger hunger drive in females [194]. Consistent with this, epidemiological studies report more pronounced links between late eating and inflammation in women: one cohort found that each 10% increase in daily calories consumed after 17:00 raised CRP levels by 3% in women, but not in men [32]. It appears that women are more vulnerable to the pro-inflammatory and insulin-resistant effects of nocturnal eating, possibly due to estrogen’s interactions with clock-controlled metabolic pathways.

## 7. Influence of Lifestyle Factors on Circadian Health

### 7.1. Sleep Quality and Quantity

Sleep is a fundamental component of circadian health. The sleep–wake cycle is one of the most prominent manifestations of circadian rhythms. Adequate sleep supports cognitive function, emotional regulation, immune response, and metabolic processes [195,196,197,198]. Disruptions in sleep patterns can lead to misalignment of circadian rhythms, resulting in various health issues. Insufficient or poor-quality sleep has been linked to numerous adverse health outcomes. Chronic sleep deprivation can impair glucose metabolism, increase appetite, and elevate the risk of obesity and T2DM. It also affects cardiovascular health by increasing blood pressure and promoting inflammation. Moreover, sleep disturbances can exacerbate mental health conditions such as depression and anxiety [196,199].

Several lifestyle factors influence sleep quality. Caffeine and alcohol consumption can delay sleep onset and disrupt sleep architecture [200,201,202,203]. Exposure to blue light from screens before bedtime suppresses melatonin production, leading to poor sleep. Irregular sleep schedules desynchronize the internal clock, while stress and anxiety elevate cortisol levels, further impairing sleep. Strategies to enhance sleep quality include maintaining a consistent sleep schedule, creating a conducive sleep environment, limiting screen time before bed, managing stress through relaxation techniques, and monitoring dietary intake [204,205].

### 7.2. Physical Activity Timing

Physical activity serves as a potent zeitgeber capable of influencing circadian rhythms. Regular exercise can help synchronize the internal clock, improving sleep quality and overall health. The timing of physical activity can differentially affect circadian rhythms. Morning exercise can advance the circadian phase and is beneficial for individuals with delayed sleep phase disorders. Afternoon exercise aligns with the body’s peak performance time, enhancing workout efficiency and circadian alignment. Evening exercise, depending on intensity, may delay sleep onset, though moderate activities like yoga may be less disruptive [206,207,208].

Exercise influences circadian rhythms through temperature regulation, hormonal effects, and gene expression. Physical activity increases core body temperature, which subsequently drops post-exercise, facilitating sleep onset. Exercise also affects hormones like cortisol and melatonin and modulates the expression of clock genes in peripheral tissues. To optimize exercise benefits on circadian health, individuals should maintain consistency, align exercise timing with personal schedules, and combine morning workouts with natural light exposure [209,210].

### 7.3. Stress and Social Rhythms

Chronic stress can disrupt circadian rhythms by affecting the hypothalamic–pituitary–adrenal (HPA) axis. Elevated evening cortisol levels can delay sleep onset and reduce sleep quality. Stress-induced circadian misalignment is associated with metabolic disorders, cardiovascular diseases, and mood disorders [184]. Social cues, such as meal times, work schedules, and social interactions, act as secondary zeitgebers. Irregular social rhythms, common among shift workers and individuals with erratic schedules, can desynchronize internal clocks and impair circadian health [57,184,211].

### 7.4. Light Exposure (Natural vs. Artificial)

Light is the primary zeitgeber for the human circadian system. Exposure to natural light during the day promotes alertness and synchronizes the internal clock with the external environment. Natural light exposure, particularly in the morning, enhances mood, improves sleep quality, and reinforces circadian rhythms. In contrast, artificial light at night, especially blue light from electronic screens, suppresses melatonin production and delays sleep onset [212,213,214,215].

Excessive artificial lighting in urban environments, or light pollution, can lead to chronic circadian disruption. To mitigate these effects, individuals should maximize daylight exposure, particularly in the morning, limit exposure to artificial light in the evening, and ensure the sleep environment is dark. The use of blackout curtains and reduced screen time before bed are practical measures for maintaining circadian alignment [214,216,217].

## 8. Conclusions

The current review underscores the significant role of chrononutrition in shaping energy balance, weight regulation, and overall metabolic health. An expanding body of research demonstrates that not only what we eat, but when we eat, profoundly impacts lipid metabolism, glucose homeostasis, and body composition. Key findings reveal that meal timing, particularly aligning food intake with circadian rhythms—such as through TRE or early eating windows—can enhance metabolic outcomes independently of caloric intake or macronutrient composition. Specifically, consuming meals earlier in the day has a link with improved lipid profiles, reduced insulin resistance, and lower body fat percentage, supporting the emerging concept of circadian-aligned eating.

The timing of meals is emerging as a promising and adjustable lifestyle factor that could support conventional dietary approaches in preventing and managing metabolic disorders. Unlike rigid dietary restrictions, optimizing meal timing offers a feasible and sustainable behavioral intervention with minimal cost and wide applicability. Its potential for public health impact is especially compelling given the increasing global burden of obesity, T2DM, and cardiovascular disease.

To fully harness the benefits of chrononutrition, future strategies must adopt an integrated approach—combining optimal diet composition, consistent meal timing, and the maintenance of circadian health through regular sleep–wake cycles and light exposure. Such a holistic framework recognizes the interplay between biological rhythms and metabolic regulation, providing a more personalized and effective model of nutritional intervention.

However, current evidence is still limited by short study durations, small sample sizes, and heterogeneity in protocols. Long-term, large-scale randomized clinical trials are needed to establish causality, define optimal timing windows, and assess the sustainability and safety of meal-timing interventions across diverse populations. Additionally, mechanistic studies exploring human circadian biology—particularly at the molecular and hormonal levels—are essential to elucidate how temporal patterns of eating interact with endogenous clocks to influence metabolic pathways.

In conclusion, while preliminary data are promising, advancing chrononutrition from theory to clinical practice will require robust scientific inquiry and multidisciplinary collaboration. Only through such efforts can we unlock its full potential for improving metabolic health and preventing chronic disease.

## Figures and Tables

**Table 1:** Comparative table of the metabolic effects of TRE.

Study	Design and Population	TRE Intervention	Key Metabolic Outcomes	Observations
Réda et al. (2020) [150]	Systematic review of 23 human studies	4–12 h eating windows without caloric restriction	Average 3% reduction in body weight and fat loss; improved glucose, lipid levels, and blood pressure, independent of weight loss	Benefits attributed to circadian rhythm realignment
Kamarul Zaman et al. (2023) [151]	Systematic review and meta-analysis of studies with varying eating window durations	8 h and >8 h windows	8 h windows showed significant weight loss (−1.18 kg); >8 h windows showed no significant difference	Interventions longer than 12 weeks were associated with greater benefits
Silva et al. (2025) [152]	Systematic review and meta-analysis of studies in overweight or obese adults	Restricted eating windows	Increased hunger; variable effects on weight loss and body composition	Highlights the need to consider adherence and impact on appetite
Hays et al. (2025) [153]	Systematic review and meta-analysis of 15 studies with 338 participants combining TRE and exercise	TRE combined with exercise (aerobic, resistance, or both)	Significant reduction in fat mass (effect size: −0.20) and body fat percentage (effect size: −0.23); no significant changes in fat-free mass	TRE combined with exercise may be more effective for reducing fat while preserving muscle mass

## References

[1] Simancas-Racines D., Román-Galeano N.M., Verde L., Annunziata G., Marchetti M., Matos A., Campuzano-Donoso M., Reytor-González C., Muscogiuri G., Barrea L. (2025). Targeting Cytokine Dysregulation in Psoriasis: The Role of Dietary Interventions in Modulating the Immune Response. Int. J. Mol. Sci..

[2] Reytor-González C., Annunziata G., Campuzano-Donoso M., Morales-López T., Basantes-Tituaña C., Fascì-Spurio F., Verde L., Muscogiuri G., Barrea L., Frias-Toral E. (2025). Endocrinologist’s crucial role in metabolic dysfunction-associated steatotic liver disease: A comprehensive review. Minerva Endocrinol..

[3] Barrea L., Frias-Toral E., Pugliese G., Garcia-Velasquez E., Carignano M.D.L.A., Savastano S., Colao A., Muscogiuri G. (2021). Vitamin D in obesity and obesity-related diseases: An overview. Minerva Endocrinol..

[4] Frias-Toral E., Garcia-Velasquez E., de Los Angeles Carignano M., Rodriguez-Veintimilla D., Alvarado-Aguilera I., Bautista-Litardo N. (2022). Polycystic ovary syndrome and obesity: Clinical aspects and nutritional management. Minerva Endocrinol..

[5] Frias-Toral E., Ceriani F., Carriel-Mancilla J., Ramos A. (2025). Editorial: Understanding obesity to determine the best therapeutic option: From lifestyle interventions to therapies. Front. Nutr..

[6] Simancas-Racines D., Annunziata G., Verde L., Fascì-Spurio F., Reytor-González C., Muscogiuri G., Frias-Toral E., Barrea L. (2025). Nutritional Strategies for Battling Obesity-Linked Liver Disease: The Role of Medical Nutritional Therapy in Metabolic Dysfunction-Associated Steatotic Liver Disease (MASLD) Management. Curr. Obes. Rep..

[7] Verde L., Frias-Toral E., Cardenas D. (2023). Editorial: Environmental factors implicated in obesity. Front. Nutr..

[8] Suárez R., Chapela S.P., Álvarez-Córdova L., Bautista-Valarezo E., Sarmiento-Andrade Y., Verde L., Frias-Toral E., Sarno G. (2023). Epigenetics in Obesity and Diabetes Mellitus: New Insights. Nutrients.

[9] Reytor-González C., Zambrano A.K., Frias-Toral E., Campuzano-Donoso M., Simancas-Racines D. (2025). Mediterranean diet and breast cancer: A narrative review. Medwave.

[10] Boutari C., Mantzoros C.S. (2022). A 2022 update on the epidemiology of obesity and a call to action: As its twin COVID-19 pandemic appears to be receding, the obesity and dysmetabolism pandemic continues to rage on. Metabolism..

[11] Emmerich S.D., Fryar C.D., Stierman B., Ogden C.L. (2024). Obesity and Severe Obesity Prevalence in Adults: United States, August 2021-August 2023. NCHS Data Brief.

[12] Muscogiuri G., Zanata I., Barrea L., Cozzolino A., Filice E., Messina E., Colao A., Faggiano A. (2023). A practical nutritional guideline to manage neuroendocrine neoplasms through chronotype and sleep. Crit. Rev. Food Sci. Nutr..

[13] Docimo A., Verde L., Barrea L., Vetrani C., Memoli P., Accardo G., Colella C., Nosso G., Orio M., Renzullo A. (2023). Type 2 Diabetes: Also a “Clock Matter”?. Nutrients.

[14] Reytor-González C., Zambrano A.K., Montalvan M., Frias-Toral E., Simancas-Racines A., Simancas-Racines D. (2024). Adherence to the Mediterranean Diet and its association with gastric cancer: Health benefits from a Planeterranean perspective. J. Transl. Med..

[15] Zambrano A.K., Cadena-Ullauri S., Ruiz-Pozo V.A., Tamayo-Trujillo R., Paz-Cruz E., Guevara-Ramírez P., Frias-Toral E., Simancas-Racines D. (2024). Impact of fundamental components of the Mediterranean diet on the microbiota composition in blood pressure regulation. J. Transl. Med..

[16] Barrea L., Verde L., Savastano S., Colao A., Muscogiuri G. (2023). Adherence to Mediterranean Diet: Any Association with NAFLD?. Antioxidants.

[17] Vetrani C., Verde L., Colao A., Barrea L., Muscogiuri G. (2023). The Mediterranean Diet: Effects on Insulin Resistance and Secretion in Individuals with Overweight or Obesity. Nutrients.

[18] Godos J., Scazzina F., Paternò Castello C., Giampieri F., Quiles J.L., Briones Urbano M., Battino M., Galvano F., Iacoviello L., de Gaetano G. (2024). Underrated aspects of a true Mediterranean diet: Understanding traditional features for worldwide application of a “Planeterranean” diet. J. Transl. Med..

[19] Muscogiuri G., Verde L., Sulu C., Katsiki N., Hassapidou M., Frias-Toral E., Cucalón G., Pazderska A., Yumuk V.D., Colao A. (2022). Mediterranean Diet and Obesity-related Disorders: What is the Evidence?. Curr. Obes. Rep..

[20] Suárez R., Bautista-Valarezo E., Matos A., Calderón P., Fascì-Spurio F., Castano-Jimenez J., Zambrano-Villacres R., Sarno G., Frias-Toral E. (2025). Obesity and nutritional strategies: Advancing prevention and management through evidence-based approaches. Food Agric. Immunol..

[21] Reytor-González C., Simancas-Racines D., Campuzano-Donoso M., Castano Jimenez J., Román-Galeano N.M., Sarno G., Frias-Toral E. (2025). Harnessing nutrition to combat MASLD: A comprehensive guide to food-based therapeutic strategies. Food Agric. Immunol..

[22] Chapela S., Locatelli J., Saettone F., Forte C.A., Memoli P., Cucalon G., Ceriani F., Sarno G., Coppola L., Frias-Toral E. (2025). The role of nutrition in cancer prevention: The effect of dietary patterns, bioactive compounds, and metabolic pathways on cancer development. Food Agric. Immunol..

[23] Zambrano A.K., Cadena-Ullauri S., Guevara-Ramírez P., Frias-Toral E., Ruiz-Pozo V.A., Paz-Cruz E., Tamayo-Trujillo R., Chapela S., Montalván M., Sarno G. (2023). The Impact of a Very-Low-Calorie Ketogenic Diet in the Gut Microbiota Composition in Obesity. Nutrients.

[24] Chapela S.P., Simancas-Racines A., Ceriani F., Martinuzzi A.L.N., Russo M.P., Zambrano A.K., Simancas-Racines D., Verde L., Muscogiuri G., Katsanos C.S. (2024). Obesity and Obesity-Related Thyroid Dysfunction: Any Potential Role for the Very Low-Calorie Ketogenic Diet (VLCKD)?. Curr. Nutr. Rep..

[25] Barrea L., Caprio M., Camajani E., Verde L., Elce A., Frias-Toral E., Ceriani F., Cucalón G., Garcia-Velasquez E., El Ghoch M. (2023). Clinical and nutritional management of very-low-calorie ketogenic diet (VLCKD) in patients with psoriasis and obesity: A practical guide for the nutritionist. Crit. Rev. Food Sci. Nutr..

[26] Barrea L., Caprio M., Tuccinardi D., Moriconi E., Di Renzo L., Muscogiuri G., Colao A., Savastano S. (2022). Could ketogenic diet “starve” cancer? Emerging evidence. Crit. Rev. Food Sci. Nutr..

[27] Hall K.D., Farooqi I.S., Friedman J.M., Klein S., Loos R.J.F., Mangelsdorf D.J., O’Rahilly S., Ravussin E., Redman L.M., Ryan D.H. (2022). The energy balance model of obesity: Beyond calories in, calories out. Am. J. Clin. Nutr..

[28] Theodorakis N., Kreouzi M., Pappas A., Nikolaou M. (2024). Beyond Calories: Individual Metabolic and Hormonal Adaptations Driving Variability in Weight Management—A State-of-the-Art Narrative Review. Int. J. Mol. Sci..

[29] Contreras F., Al-Najim W., le Roux C.W. (2024). Health Benefits Beyond the Scale: The Role of Diet and Nutrition During Weight Loss Programmes. Nutrients.

[30] Guarneiri L.L., Adams C.G., Garcia-Jackson B., Koecher K., Wilcox M.L., Maki K.C. (2024). Effects of Varying Protein Amounts and Types on Diet-Induced Thermogenesis: A Systematic Review and Meta-Analysis. Adv. Nutr..

[31] Bojarczuk A., Egorova E.S., Dzitkowska-Zabielska M., Ahmetov I.I. (2024). Genetics of Exercise and Diet-Induced Fat Loss Efficiency: A Systematic Review. J. Sports Sci. Med..

[32] Raji O.E., Kyeremah E.B., Sears D.D., St-Onge M.P., Makarem N. (2024). Chrononutrition and Cardiometabolic Health: An Overview of Epidemiological Evidence and Key Future Research Directions. Nutrients.

[33] Verde L., Di Lorenzo T., Savastano S., Colao A., Barrea L., Muscogiuri G. (2024). Chrononutrition in type 2 diabetes mellitus and obesity: A narrative review. Diabetes Metab. Res. Rev..

[34] Muscogiuri G. (2024). The timing of energy intake. Proc. Nutr. Soc..

[35] Verde L., Docimo A., Chirico G., Savastano S., Colao A., Barrea L., Muscogiuri G. (2023). How Fast Do “Owls” and “Larks” Eat?. Nutrients.

[36] Fagiani F., Di Marino D., Romagnoli A., Travelli C., Voltan D., Mannelli L.D.C., Racchi M., Govoni S., Lanni C. (2022). Molecular regulations of circadian rhythm and implications for physiology and diseases. Signal Transduct. Target. Ther..

[37] Mortimer T., Smith J.G., Muñoz-Cánoves P., Benitah S.A. (2025). Circadian clock communication during homeostasis and ageing. Nat. Rev. Mol. Cell Biol..

[38] Miro C., Docimo A., Barrea L., Verde L., Cernea S., Sojat A.S., Marina L.V., Docimo G., Colao A., Dentice M. (2023). “Time” for obesity-related cancer: The role of the circadian rhythm in cancer pathogenesis and treatment. Semin. Cancer Biol..

[39] Mentzelou M., Papadopoulou S.K., Psara E., Voulgaridou G., Pavlidou E., Androutsos O., Giaginis C. (2024). Chrononutrition in the Prevention and Management of Metabolic Disorders: A Literature Review. Nutrients.

[40] Zinna L., Verde L., Di Tolla M.F., Barrea L., Parascandolo A., D’Alterio F., Colao A., Formisano P., D’Esposito V., Muscogiuri G. (2025). Chronodisruption enhances inflammatory cytokine release from visceral adipose tissue in obesity. J. Transl. Med..

[41] Peters B., Vahlhaus J., Pivovarova-Ramich O. (2024). Meal timing and its role in obesity and associated diseases. Front. Endocrinol..

[42] Godos J., Currenti W., Ferri R., Lanza G., Caraci F., Frias-Toral E., Guglielmetti M., Ferraris C., Lipari V., Carvajal Altamiranda S. (2025). Chronotype and Cancer: Emerging Relation Between Chrononutrition and Oncology from Human Studies. Nutrients.

[43] Myung J., Vitet H., Truong V.H., Ananthasubramaniam B. (2025). The role of the multiplicity of circadian clocks in mammalian systems. Sleep Med..

[44] Evans J., Silver R., Pfaff D.W., Volkow N.D., Rubenstein J. (2020). The Suprachiasmatic Nucleus and the Circadian Timekeeping System of the Body.

[45] Page A.J. (2021). Gastrointestinal vagal afferents and food intake: Relevance of circadian rhythms. Nutrients.

[46] Reick M., Garcia J.A., Dudley C., McKnight S.L. (2001). NPAS2: An analog of clock operative in the mammalian forebrain. Science.

[47] Gekakis N., Staknis D., Nguyen H.B., Davis F.C., Wilsbacher L.D., King D.P., Takahashi J.S., Weitz C.J. (1998). Role of the CLOCK Protein in the Mammalian Circadian Mechanism. Science.

[48] Bunger M.K., Wilsbacher L.D., Moran S.M., Clendenin C., Radcliffe L.A., Hogenesch J.B., Simon M.C., Takahashi J.S., Bradfield C.A. (2000). Mop3 Is an Essential Component of the Master Circadian Pacemaker in Mammals. Cell.

[49] Yuan V.G. (2025). Rhythms in Remodeling: Posttranslational Regulation of Bone by the Circadian Clock. Biomedicines.

[50] Gnocchi D., Bruscalupi G. (2017). Circadian Rhythms and Hormonal Homeostasis: Pathophysiological Implications. Biology.

[51] Neumann A.-M., Schmidt C.X., Brockmann R.M., Oster H. (2019). Circadian regulation of endocrine systems. Auton. Neurosci..

[52] Meléndez-Fernández O.H., Liu J.A., Nelson R.J. (2023). Circadian Rhythms Disrupted by Light at Night and Mistimed Food Intake Alter Hormonal Rhythms and Metabolism. Int. J. Mol. Sci..

[53] Aykan U., Güvel M.C., Paykal G., Uluoglu C. (2023). Neuropharmacologic modulation of the melatonergic system. Explor. Neurosci..

[54] Amaral F.G.D., Cipolla-Neto J. (2018). A brief review about melatonin, a pineal hormone. Arch. Endocrinol. Metab..

[55] Andreadi A., Andreadi S., Todaro F., Ippoliti L., Bellia A., Magrini A., Chrousos G.P., Lauro D. (2025). Modified Cortisol Circadian Rhythm: The Hidden Toll of Night-Shift Work. Int. J. Mol. Sci..

[56] Spencer R.L., Chun L.E., Hartsock M.J., Woodruff E.R. (2017). Glucocorticoid hormones are both a major circadian signal and major stress signal. Front. Neuroendocrinol..

[57] Chan S., Debono M. (2010). Replication of cortisol circadian rhythm: New advances in hydrocortisone replacement therapy. Ther. Adv. Endocrinol. Metab..

[58] Wang L., Hu L., Wang X., Geng Z., Wan M., Hao J., Liu H., Fan Y., Xu T., Li Z. (2024). Long non-coding RNA LncCplx2 regulates glucose homeostasis and pancreatic β cell function. Mol. Metab..

[59] Seshadri N., Doucette C.A. (2021). Circadian Regulation of the Pancreatic Beta Cell. Endocrinology.

[60] Qian J., Scheer F.A.J.L. (2016). Circadian System and Glucose Metabolism: Implications for Physiology and Disease. Trends Endocrinol. Metab..

[61] Lee D.Y., Jung I., Park S.Y., Yu J.H., Seo J.A., Kim K.J., Kim N.H., Yoo H.J., Kim S.G., Choi K.M. (2024). Attention to Innate Circadian Rhythm and the Impact of Its Disruption on Diabetes. Diabetes Metab. J..

[62] Ruddick-Collins L.C., Morgan P.J., Johnstone A.M. (2020). Mealtime: A circadian disruptor and determinant of energy balance?. J. Neuroendocrinol..

[63] Farhadipour M., Depoortere I. (2021). The function of gastrointestinal hormones in obesity—Implications for the regulation of energy intake. Nutrients.

[64] Miller G.D. (2019). Appetite Regulation: Hormones, Peptides, and Neurotransmitters and Their Role in Obesity. Am. J. Lifestyle Med..

[65] Ribeiro F.M., Arnaldo L., Milhomem L.P., Aguiar S.S., Franco O.L. (2025). The intricate relationship between circadian rhythms and gastrointestinal peptides in obesity. Peptides.

[66] Van Drunen R., Eckel-Mahan K. (2021). Circadian Rhythms of the Hypothalamus: From Function to Physiology. Clocks Sleep.

[67] Schmal C., Herzel H., Myung J. (2020). Clocks in the Wild: Entrainment to Natural Light. Front. Physiol..

[68] Zele A.J., Feigl B., Smith S.S., Markwell E.L., Dryer S. (2011). The circadian response of intrinsically photosensitive retinal ganglion cells. PLoS ONE.

[69] Foster R.G., Hughes S., Peirson S.N. (2020). Circadian Photoentrainment in Mice and Humans. Biology.

[70] Hunter A.L., Bechtold D.A. (2025). The metabolic significance of peripheral tissue clocks. Commun. Biol..

[71] Rodrigues L.G.F., de Araujo L.D., Roa S.L.R., Bueno A.C., Uchoa E.T., Antunes-Rodrigues J., Moreira A.C., Elias L.L.K., de Castro M., Martins C.S. (2021). Restricted feeding modulates peripheral clocks and nutrient sensing pathways in rats. Arch. Endocrinol. Metab..

[72] Manella G., Sabath E., Aviram R., Dandavate V., Ezagouri S., Golik M., Adamovich Y., Asher G. (2021). The liver-clock coordinates rhythmicity of peripheral tissues in response to feeding. Nat. Metab..

[73] Vollmers C., Gill S., DiTacchio L., Pulivarthy S.R., Le H.D., Panda S. (2009). Time of feeding and the intrinsic circadian clock drive rhythms in hepatic gene expression. Proc. Natl. Acad. Sci. USA.

[74] Costa R., Mangini C., Domenie E.D., Zarantonello L., Montagnese S. (2023). Circadian rhythms and the liver. Liver Int..

[75] Pickel L., Sung H.-K. (2020). Feeding Rhythms and the Circadian Regulation of Metabolism. Front. Nutr..

[76] Xie Y., Tang Q., Chen G., Xie M., Yu S., Zhao J., Chen L. (2019). New insights into the circadian rhythm and its related diseases. Front. Physiol..

[77] Hamaguchi Y., Tahara Y., Kuroda H., Haraguchi A., Shibata S. (2015). Entrainment of mouse peripheral circadian clocks to <24 h feeding/fasting cycles under 24 h light/dark conditions. Sci. Rep..

[78] Zhang Y., Li Y., Noya S.B., Sehgal A. (2024). The microbiome interacts with the circadian clock and dietary composition to regulate metabolite cycling in the gut. BioExiv.

[79] Garcia-Rios A., Ordovas J.M. (2022). Chronodisruption and cardiovascular disease. Clin. Investig. Arterioscler. Publ. Of. Soc. Esp. Arterioscler..

[80] Ekiz Erim S., Sert H. (2023). The relationship between chronotype and obesity: A systematic review. Chronobiol. Int..

[81] Muscogiuri G., Barrea L., Caprio M., Ceriani F., Chavez A.O., El Ghoch M., Frias-Toral E., Mehta R.J., Mendez V., Paschou S.A. (2022). Nutritional guidelines for the management of insulin resistance. Crit. Rev. Food Sci. Nutr..

[82] Blasiak A., Gundlach A.L., Hess G., Lewandowski M.H. (2017). Interactions of circadian rhythmicity, stress and orexigenic neuropeptide systems: Implications for food intake control. Front. Neurosci..

[83] Kalra S.P., Bagnasco M., Otukonyong E.E., Dube M.G., Kalra P.S. (2003). Rhythmic, reciprocal ghrelin and leptin signaling: New insight in the development of obesity. Regul. Pept..

[84] Grosjean E., Simonneaux V., Challet E. (2023). Reciprocal Interactions between Circadian Clocks, Food Intake, and Energy Metabolism. Biology.

[85] Blancas-Velazquez A., Mendoza J., Garcia A.N., la Fleur S.E. (2017). Diet-induced obesity and circadian disruption of feeding behavior. Front. Neurosci..

[86] Gallop M.R., Tobin S.Y., Chaix A. (2023). Finding balance: Understanding the energetics of time-restricted feeding in mice. Obesity.

[87] Begemann K., Heyde I., Witt P., Inderhees J., Leinweber B., Koch C.E., Jöhren O., Oelkrug R., Liskiewicz A., Müller T.D. (2023). Rest phase snacking increases energy resorption and weight gain in male mice. Mol. Metab..

[88] Arble D.M., Bass J., Laposky A.D., Vitaterna M.H., Turek F.W. (2009). Circadian timing of food intake contributes to weight gain. Obesity.

[89] Theodorakis N., Nikolaou M. (2025). The Human Energy Balance: Uncovering the Hidden Variables of Obesity. Diseases.

[90] Poggiogalle E., Jamshed H., Peterson C.M. (2018). Circadian regulation of glucose, lipid, and energy metabolism in humans. Metabolism.

[91] Zhao E., Tait C., Minacapelli C.D., Catalano C., Rustgi V.K. (2022). Circadian Rhythms, the Gut Microbiome, and Metabolic Disorders. Gastro Hep Adv..

[92] Zhang Z., Wang S., Gao L. (2025). Circadian rhythm, glucose metabolism and diabetic complications: The role of glucokinase and the enlightenment on future treatment. Front. Physiol..

[93] Davis R., Bonham M.P., Nguo K., Huggins C.E. (2020). Glycaemic response at night is improved after eating a high protein meal compared with a standard meal: A cross-over study. Clin. Nutr..

[94] Leung G.K.W., Huggins C.E., Ware R.S., Bonham M.P. (2020). Time of day difference in postprandial glucose and insulin responses: Systematic review and meta-analysis of acute postprandial studies. Chronobiol. Int..

[95] Mason I.C., Qian J., Adler G.K., Scheer F.A.J.L. (2020). Impact of circadian disruption on glucose metabolism: Implications for type 2 diabetes. Diabetologia.

[96] Chan K., Wong F.S., Pearson J.A. (2022). Circadian rhythms and pancreas physiology: A review. Front. Endocrinol..

[97] Laudet V. (2008). Nuclear receptors: At the heart of the biological crosstalk between metabolism and circadian rhythm. Expert Rev. Endocrinol. Metab..

[98] Hunter A.L., Ray D.W. (2019). Circadian Clock Regulation of Hepatic Energy Metabolism Regulatory Circuits. Biology.

[99] Liu M., Zhang Z., Chen Y., Feng T., Zhou Q., Tian X. (2023). Circadian clock and lipid metabolism disorders: A potential therapeutic strategy for cancer. Front. Endocrinol..

[100] Paoli A. (2025). The Influence of Physical Exercise, Ketogenic Diet, and Time-Restricted Eating on De Novo Lipogenesis: A Narrative Review. Nutrients.

[101] Shen S., Shen M., Kuang L., Yang K., Wu S., Liu X., Wang Y., Wang Y. (2024). SIRT1/SREBPs-mediated regulation of lipid metabolism. Pharmacol. Res..

[102] Guo Y., Livelo C., Melkani G.C. (2023). Time-restricted feeding regulates lipid metabolism under metabolic challenges. Bioessays.

[103] Petrenko V., Sinturel F., Riezman H., Dibner C. (2023). Lipid metabolism around the body clocks. Prog. Lipid Res..

[104] Adamovich Y., Rousso-Noori L., Zwighaft Z., Neufeld-Cohen A., Golik M., Kraut-Cohen J., Wang M., Han X., Asher G. (2014). Circadian clocks and feeding time regulate the oscillations and levels of hepatic triglycerides. Cell Metab..

[105] Yan L., Rust B.M., Palmer D.G. (2024). Time-restricted feeding restores metabolic flexibility in adult mice with excess adiposity. Front. Nutr..

[106] Elena C., Flavia L., Davide M., Mariaignazia C., Chandra M., Orietta G., Elena G., Mikiko W., Stefania M., Lucio G. (2025). Eating behavior patterns, metabolic parameters and circulating oxytocin levels in patients with obesity: An exploratory study. Eat. Weight Disord. Stud. Anorex. Bulim. Obes..

[107] da Cunha N.B., Teixeira G.P., Rinaldi A.E.M., Azeredo C.M., Crispim C.A. (2023). Late meal intake is associated with abdominal obesity and metabolic disorders related to metabolic syndrome: A chrononutrition approach using data from NHANES 2015–2018. Clin. Nutr..

[108] Gonzalez J.T., Batterham A.M., Atkinson G., Thompson D. (2023). Perspective: Is the Response of Human Energy Expenditure to Increased Physical Activity Additive or Constrained?. Adv. Nutr..

[109] Careau V., Halsey L.G., Pontzer H., Ainslie P.N., Andersen L.F., Anderson L.J., Arab L., Baddou I., Bedu-Addo K., Blaak E.E. (2021). Energy compensation and adiposity in humans. Curr. Biol..

[110] Westerterp K.R. (2004). Diet induced thermogenesis. Nutr. Metab..

[111] Zitting K.-M., Vujovic N., Yuan R.K., Isherwood C.M., Medina J.E., Wang W., Buxton O.M., Williams J.S., Czeisler C.A., Duffy J.F. (2018). Human Resting Energy Expenditure Varies with Circadian Phase. Curr. Biol..

[112] Shaw E., Leung G.K.W., Jong J., Coates A.M., Davis R., Blair M., Huggins C.E., Dorrian J., Banks S., Kellow N.J. (2019). The Impact of Time of Day on Energy Expenditure: Implications for Long-Term Energy Balance. Nutrients.

[113] Refinetti R. (2020). Circadian rhythmicity of body temperature and metabolism. Temperature.

[114] Richter J., Herzog N., Janka S., Baumann T., Kistenmacher A., Oltmanns K.M. (2020). Twice as High Diet-Induced Thermogenesis After Breakfast vs Dinner on High-Calorie as Well as Low-Calorie Meals. J. Clin. Endocrinol. Metab..

[115] Potter G.D.M., Cade J.E., Grant P.J., Hardie L.J. (2016). Nutrition and the circadian system. Br. J. Nutr..

[116] Jakubowicz D., Matz Y., Landau Z., Rosenblum R.C., Twito O., Wainstein J., Tsameret S. (2024). Interaction Between Early Meals (Big-Breakfast Diet), Clock Gene mRNA Expression, and Gut Microbiome to Regulate Weight Loss and Glucose Metabolism in Obesity and Type 2 Diabetes. Int. J. Mol. Sci..

[117] Zhang X., Xiao J., Jiang M., Phillips C.J.C., Shi B. (2025). Thermogenesis and Energy Metabolism in Brown Adipose Tissue in Animals Experiencing Cold Stress. Int. J. Mol. Sci..

[118] Yuko O.O., Saito M. (2021). Brown fat as a regulator of systemic metabolism beyond thermogenesis. Diabetes Metab. J..

[119] Saito M., Okamatsu-Ogura Y. (2023). Thermogenic Brown Fat in Humans: Implications in Energy Homeostasis, Obesity and Metabolic Disorders. World J. Mens Health.

[120] Basu R., Flak J.N. (2025). Hypothalamic neural circuits regulating energy expenditure. Vitam. Horm..

[121] Tran L.T., Park S., Kim S.K., Lee J.S., Kim K.W., Kwon O. (2022). Hypothalamic control of energy expenditure and thermogenesis. Exp. Mol. Med..

[122] Qi Y., Lee N.J., Ip C.K., Enriquez R., Tasan R., Zhang L., Herzog H. (2023). Agrp-negative arcuate NPY neurons drive feeding under positive energy balance via altering leptin responsiveness in POMC neurons. Cell Metab..

[123] Vohra M.S., Benchoula K., Serpell C.J., Hwa W.E. (2022). AgRP/NPY and POMC neurons in the arcuate nucleus and their potential role in treatment of obesity. Eur. J. Pharmacol..

[124] Jais A., Brüning J.C. (2022). Arcuate Nucleus-Dependent Regulation of Metabolism-Pathways to Obesity and Diabetes Mellitus. Endocr. Rev..

[125] Begemann K., Rawashdeh O., Olejniczak I., Pilorz V., de Assis L.V.M., Osorio-Mendoza J., Oster H. (2025). Endocrine regulation of circadian rhythms. npj Biol. Timing Sleep.

[126] Kalsbeek A., Scheer F.A., Perreau-Lenz S., La Fleur S.E., Yi C.-X., Fliers E., Buijs R.M. (2011). Circadian disruption and SCN control of energy metabolism. FEBS Lett..

[127] Starnes A.N., Jones J.R. (2023). Inputs and Outputs of the Mammalian Circadian Clock. Biology.

[128] BaHammam A.S., Pirzada A. (2023). Timing Matters: The Interplay between Early Mealtime, Circadian Rhythms, Gene Expression, Circadian Hormones, and Metabolism—A Narrative Review. Clocks Sleep.

[129] de Assis L.V.M., Oster H. (2021). The circadian clock and metabolic homeostasis: Entangled networks. Cell. Mol. Life Sci..

[130] Greco C.M., Sassone-Corsi P. (2019). Circadian blueprint of metabolic pathways in the brain. Nat. Rev. Neurosci..

[131] Obradovic M., Sudar-Milovanovic E., Soskic S., Essack M., Arya S., Stewart A.J., Gojobori T., Isenovic E.R. (2021). Leptin and Obesity: Role and Clinical Implication. Front. Endocrinol..

[132] Plano S.A., Casiraghi L.P., García Moro P., Paladino N., Golombek D.A., Chiesa J.J. (2017). Circadian and Metabolic Effects of Light: Implications in Weight Homeostasis and Health. Front. Neurol..

[133] Liu J., Yi P., Liu F. (2023). The Effect of Early Time-Restricted Eating vs Later Time-Restricted Eating on Weight Loss and Metabolic Health. J. Clin. Endocrinol. Metab..

[134] Yu Z., Ueda T. (2025). Early Time-Restricted Eating Improves Weight Loss While Preserving Muscle: An 8-Week Trial in Young Women. Nutrients.

[135] Jamshed H., Steger F.L., Bryan D.R., Richman J.S., Warriner A.H., Hanick C.J., Martin C.K., Salvy S.-J., Peterson C.M. (2022). Effectiveness of Early Time-Restricted Eating for Weight Loss, Fat Loss, and Cardiometabolic Health in Adults With Obesity: A Randomized Clinical Trial. JAMA Intern. Med..

[136] de Goede P., Foppen E., Ritsema W.I.G.R., Korpel N.L., Yi C.-X., Kalsbeek A. (2019). Time-Restricted Feeding Improves Glucose Tolerance in Rats, but Only When in Line With the Circadian Timing System. Front. Endocrinol..

[137] Yang Y., Liu D. (2024). Impacts of time-restricted feeding on middle-aged and old mice with obesity. J. Physiol..

[138] Gibney M.J., Barr S.I., Bellisle F., Drewnowski A., Fagt S., Livingstone B., Masset G., Varela Moreiras G., Moreno L.A., Smith J. (2018). Breakfast in Human Nutrition: The International Breakfast Research Initiative. Nutrients.

[139] Kurtgil S., Pekcan A.G. (2023). Determination of breakfast habits, food pattern and quality among adults. Med. J. Nutr. Metab..

[140] Xia M., Zhong Y., Peng Y., Qian C. (2024). Breakfast skipping and traits of cardiometabolic health: A mendelian randomization study. Clin. Nutr. ESPEN.

[141] Buscemi C., Randazzo C., Barile A.M., Caldarella R., Murro I., Caruso R., Colombrita P., Lombardo M., De Pergola G., Buscemi S. (2025). The impact of breakfast skipping on plasma glucose levels in non-diabetic individuals: Gender-based differences and implications. Int. J. Food Sci. Nutr..

[142] Barrea L., Frias-Toral E., Aprano S., Castellucci B., Pugliese G., Rodriguez-Veintimilla D., Vitale G., Gentilini D., Colao A., Savastano S. (2022). The clock diet: A practical nutritional guide to manage obesity through chrononutrition. Minerva Med..

[143] Ballon A., Neuenschwander M., Schlesinger S. (2019). Breakfast Skipping Is Associated with Increased Risk of Type 2 Diabetes among Adults: A Systematic Review and Meta-Analysis of Prospective Cohort Studies. J. Nutr..

[144] Ofori-Asenso R., Owen A.J., Liew D. (2019). Skipping Breakfast and the Risk of Cardiovascular Disease and Death: A Systematic Review of Prospective Cohort Studies in Primary Prevention Settings. J. Cardiovasc. Dev. Dis..

[145] Vujović N., Piron M.J., Qian J., Chellappa S.L., Nedeltcheva A., Barr D., Heng S.W., Kerlin K., Srivastav S., Wang W. (2022). Late isocaloric eating increases hunger, decreases energy expenditure, and modifies metabolic pathways in adults with overweight and obesity. Cell Metab..

[146] Ni Y., Wu L., Jiang J., Yang T., Wang Z., Ma L., Zheng L., Yang X., Wu Z., Fu Z. (2019). Late-Night Eating-Induced Physiological Dysregulation and Circadian Misalignment Are Accompanied by Microbial Dysbiosis. Mol. Nutr. Food Res..

[147] Li L., Zou J., Zhou T., Liu X., Tan D., Xiang Q., Yu R. (2025). mTOR-mediated nutrient sensing and oxidative stress pathways regulate autophagy: A key mechanism for traditional Chinese medicine to improve diabetic kidney disease. Front. Pharmacol..

[148] Chaix A., Manoogian E.N.C., Melkani G.C., Panda S. (2019). Time-Restricted Eating to Prevent and Manage Chronic Metabolic Diseases. Annu. Rev. Nutr..

[149] Fernandes-Alves D., Teixeira G.P., Guimarães K.C., Crispim C.A. (2025). Systematic Review and Meta-analysis of Randomized Clinical Trials Comparing Time-Restricted Eating with and Without Caloric Restriction for Weight Loss. Nutr. Rev..

[150] Adafer R., Messaadi W., Meddahi M., Patey A., Haderbache A., Bayen S., Messaadi N. (2020). Food timing, circadian rhythm and chrononutrition: A systematic review of time-restricted eating’s effects on human health. Nutrients.

[151] Kamarul Zaman M., Teng N.I.M.F., Kasim S.S., Juliana N., Alshawsh M.A. (2023). Effects of time-restricted eating with different eating duration on anthropometrics and cardiometabolic health: A systematic review and meta-analysis. World J. Cardiol..

[152] Silva A.D., Guimarães K.C., Oliveira R.A., Rosa D.A., Crispim C.A. (2025). Time-restricted eating increases hunger in adults with overweight and obesity: A systematic review and meta-analysis of randomized controlled studies. Nutr. Res..

[153] Hays H.M., Sefidmooye Azar P., Kang M., Tinsley G.M., Wijayatunga N.N. (2025). Effects of time-restricted eating with exercise on body composition in adults: A systematic review and meta-analysis. Int. J. Obes..

[154] Enyama Y., Takeshita Y., Tanaka T., Sako S., Kanamori T., Takamura T. (2021). Distinct effects of carbohydrate ingestion timing on glucose fluctuation and energy metabolism in patients with type 2 diabetes: A randomized controlled study. Endocr. J..

[155] Wang C., Almoosawi S., Palla L. (2019). Day-Time Patterns of Carbohydrate Intake in Adults by Non-Parametric Multi-Level Latent Class Analysis-Results from the UK National Diet and Nutrition Survey (2008/09–2015/16). Nutrients.

[156] Crabtree D.R., Buosi W., Fyfe C.L., Horgan G.W., Manios Y., Androutsos O., Giannopoulou A., Finlayson G., Beaulieu K., Meek C.L. (2020). Appetite control across the lifecourse: The acute impact of breakfast drink quantity and protein content. the full4health project. Nutrients.

[157] Nunes E.A., Colenso-Semple L., McKellar S.R., Yau T., Ali M.U., Fitzpatrick-Lewis D., Sherifali D., Gaudichon C., Tomé D., Atherton P.J. (2022). Systematic review and meta-analysis of protein intake to support muscle mass and function in healthy adults. J. Cachexia Sarcopenia Muscle.

[158] Wirth J., Hillesheim E., Brennan L. (2020). The Role of Protein Intake and its Timing on Body Composition and Muscle Function in Healthy Adults: A Systematic Review and Meta-Analysis of Randomized Controlled Trials. J. Nutr..

[159] Ezpeleta M., Cienfuegos S., Lin S., Pavlou V., Gabel K., Tussing-Humphreys L., Varady K.A. (2024). Time-restricted eating: Watching the clock to treat obesity. Cell Metab..

[160] Boyd P., O’Connor S.G., Heckman-Stoddard B.M., Sauter E.R. (2022). Time-Restricted Feeding Studies and Possible Human Benefit. JNCI Cancer Spectr..

[161] Brogi S., Tabanelli R., Puca S., Calderone V. (2024). Intermittent Fasting: Myths, Fakes and Truth on This Dietary Regimen Approach. Foods.

[162] Vasim I., Majeed C.N., DeBoer M.D. (2022). Intermittent Fasting and Metabolic Health. Nutrients.

[163] Özyildirim C., Uçar A. (2023). An alternative approach to obesity treatment: Intermittent fasting. Minerva Endocrinol..

[164] Lin S., Cienfuegos S., Ezpeleta M., Gabel K., Pavlou V., Mulas A., Chakos K., McStay M., Wu J., Tussing-Humphreys L. (2023). Time-Restricted Eating Without Calorie Counting for Weight Loss in a Racially Diverse Population: A Randomized Controlled Trial. Ann. Intern. Med..

[165] O’Connor S.G., Boyd P., Bailey C.P., Shams-White M.M., Agurs-Collins T., Hall K., Reedy J., Sauter E.R., Czajkowski S.M. (2021). Perspective: Time-Restricted Eating Compared with Caloric Restriction: Potential Facilitators and Barriers of Long-Term Weight Loss Maintenance. Adv. Nutr..

[166] O’Connor S.G., Boyd P., Bailey C.P., Nebeling L., Reedy J., Czajkowski S.M., Shams-White M.M. (2022). A qualitative exploration of facilitators and barriers of adherence to time-restricted eating. Appetite.

[167] James D.L., Hawley N.A., Mohr A.E., Hermer J., Ofori E., Yu F., Sears D.D. (2024). Impact of Intermittent Fasting and/or Caloric Restriction on Aging-Related Outcomes in Adults: A Scoping Review of Randomized Controlled Trials. Nutrients.

[168] Dai Z., Wan K., Miyashita M., Ho R.S., Zheng C., Poon E.T., Wong S.H. (2024). The Effect of Time-Restricted Eating Combined with Exercise on Body Composition and Metabolic Health: A Systematic Review and Meta-Analysis. Adv. Nutr..

[169] Chew H.S.J., Ang W.H.D., Tan Z.Y.A., Chan K.S., Lau Y. (2023). Umbrella review of time-restricted eating on weight loss, fasting blood glucose, and lipid profile. Nutr. Rev..

[170] Yao W., Wang X.-Y., Gao S., Varady K.A., Forslund S.K., Zhang M., Shi Z.-Y., Cao F., Zou B.-J., Sun M.-H. (2024). Intermittent fasting and health outcomes: An umbrella review of systematic reviews and meta-analyses of randomised controlled trials. EClinicalMedicine.

[171] Patikorn C., Roubal K., Veettil S.K., Chandran V., Pham T., Lee Y.Y., Giovannucci E.L., Varady K.A., Chaiyakunapruk N. (2021). Intermittent Fasting and Obesity-Related Health Outcomes: An Umbrella Review of Meta-analyses of Randomized Clinical Trials. JAMA Netw. Open.

[172] Regmi P., Heilbronn L.K. (2020). Time-Restricted Eating: Benefits, Mechanisms, and Challenges in Translation. iScience.

[173] Shabkhizan R., Haiaty S., Moslehian M.S., Bazmani A., Sadeghsoltani F., Saghaei Bagheri H., Rahbarghazi R., Sakhinia E. (2023). The Beneficial and Adverse Effects of Autophagic Response to Caloric Restriction and Fasting. Adv. Nutr..

[174] Mishra S., Persons P.A., Lorenzo A.M., Chaliki S.S., Bersoux S. (2023). Time-Restricted Eating and Its Metabolic Benefits. J. Clin. Med..

[175] Grosso G., Laudisio D., Frias-Toral E., Barrea L., Muscogiuri G., Savastano S., Colao A. (2022). Anti-Inflammatory Nutrients and Obesity-Associated Metabolic-Inflammation: State of the Art and Future Direction. Nutrients.

[176] Barrea L., Vetrani C., Caprio M., El Ghoch M., Frias-Toral E., Mehta R.J., Mendez V., Moriconi E., Paschou S.A., Pazderska A. (2023). Nutritional management of type 2 diabetes in subjects with obesity: An international guideline for clinical practice. Crit. Rev. Food Sci. Nutr..

[177] Lin X., Guan Y., Wu G., Huang J., Wang S. (2022). Time-restricted eating for patients with diabetes and prediabetes: A systematic review. Front. Nutr..

[178] Kyriazis I.D., Vassi E., Alvanou M., Angelakis C., Skaperda Z., Tekos F., Garikipati V.N.S., Spandidos D.A., Kouretas D. (2022). The impact of diet upon mitochondrial physiology (Review). Int. J. Mol. Med..

[179] Pradeepkiran J.A., Islam M.A., Sehar U., Reddy A.P., Vijayan M., Reddy P.H. (2025). Impact of diet and exercise on mitochondrial quality and mitophagy in Alzheimer’s disease. Ageing Res. Rev..

[180] Currenti W., Godos J., Castellano S., Caruso G., Ferri R., Caraci F., Grosso G., Galvano F. (2021). Time-restricted feeding is associated with mental health in elderly Italian adults. Chronobiol. Int..

[181] O’Neal M.A., Gutierrez N.R., Laing K.L., Manoogian E.N.C., Panda S. (2022). Barriers to adherence in time-restricted eating clinical trials: An early preliminary review. Front. Nutr..

[182] Nye K., Cherrin C., Meires J. (2024). Intermittent Fasting: Exploring Approaches, Benefits, and Implications for Health and Weight Management. J. Nurse Pract..

[183] Schrader L.A., Ronnekleiv-Kelly S.M., Hogenesch J.B., Bradfield C.A., Malecki K.M. (2024). Circadian disruption, clock genes, and metabolic health. J. Clin. Investig..

[184] Brum M.C.B., Senger M.B., Schnorr C.C., Ehlert L.R., Rodrigues T.d.C. (2022). Effect of night-shift work on cortisol circadian rhythm and melatonin levels. Sleep Sci..

[185] Azmi N.A.S.M., Juliana N., Teng N.I.M.F., Azmani S., Das S., Effendy N. (2020). Consequences of circadian disruption in shift workers on chrononutrition and their psychosocial well-being. Int. J. Environ. Res. Public Health.

[186] Hasler B.P., Soehner A.M., Clark D.B. (2015). Sleep and circadian contributions to adolescent alcohol use disorder. Alcohol.

[187] Arns M., Kooij J.J.S., Coogan A.N. (2021). Review: Identification and Management of Circadian Rhythm Sleep Disorders as a Transdiagnostic Feature in Child and Adolescent Psychiatry. J. Am. Acad. Child Adolesc. Psychiatry.

[188] Souza M.R., Neves M.E.A., Gorgulho B.M., Souza A.M., Nogueira P.S., Ferreira M.G., Rodrigues P.R.M. (2021). Breakfast skipping and cardiometabolic risk factors in adolescents: Systematic review. Rev. Saude Publica.

[189] Verde L., Barrea L., Vetrani C., Frias-Toral E., Chapela S.P., Jayawardena R., de Alteriis G., Docimo A., Savastano S., Colao A. (2022). Chronotype and Sleep Quality in Obesity: How Do They Change After Menopause?. Curr. Obes. Rep..

[190] Godos J., Grosso G., Ferri R., Caraci F., Lanza G., Al-Qahtani W.H., Caruso G., Castellano S. (2023). Mediterranean diet, mental health, cognitive status, quality of life, and successful aging in southern Italian older adults. Exp. Gerontol..

[191] Kessler K., Pivovarova-Ramich O. (2019). Meal Timing, Aging, and Metabolic Health. Int. J. Mol. Sci..

[192] Ferreira L.L., Rosatto N., Marzullo P., Bellan M. (2024). Circadian variations in the elderly: A scoping review. Chronobiol. Int..

[193] Verde L., Barrea L., Docimo A., Savastano S., Colao A., Muscogiuri G. (2023). Chronotype as a predictor of weight loss and body composition improvements in women with overweight or obesity undergoing a very low-calorie ketogenic diet (VLCKD). Clin. Nutr..

[194] Lok R., Qian J., Chellappa S.L. (2024). Sex differences in sleep, circadian rhythms, and metabolism: Implications for precision medicine. Sleep Med. Rev..

[195] Wang W., Zhao L., He Z., Zhao Y., Jiang G., Gong C., Zhang Y., Yu J., Liang T., Guo L. (2025). Decoding Multifaceted Roles of Sleep-Related Genes as Molecular Bridges in Chronic Disease Pathogenesis. Int. J. Mol. Sci..

[196] Desai D., Hirpara P., Jha H., Thaker R., Patel J., Momin A.S. (2024). Exploring the Role of Circadian Rhythms in Sleep and Recovery: A Review Article. Cureus.

[197] Barrea L., Verde L., Di Lorenzo C., Savastano S., Colao A., Muscogiuri G. (2023). Can the ketogenic diet improve our dreams? Effect of very low-calorie ketogenic diet (VLCKD) on sleep quality. J. Transl. Med..

[198] Godos J., Rosi A., Scazzina F., Bonifaz M.A.T., Giampieri F., Abdelkarim O., Ammar A., Aly M., Frias-Toral E., Pons J. (2025). Diet, Eating Habits, and Lifestyle Factors Associated with Adequate Sleep Duration in Children and Adolescents Living in 5 Mediterranean Countries: The DELICIOUS Project. Nutrients.

[199] Barrea L., Pugliese G., Frias-Toral E., Napolitano B., Laudisio D., Aprano S., Ceriani F., Savastano S., Colao A., Muscogiuri G. (2022). Is there a relationship between the ketogenic diet and sleep disorders?. Int. J. Food Sci. Nutr..

[200] O’Callaghan F., Muurlink O., Reid N. (2018). Effects of caffeine on sleep quality and daytime functioning. Risk Manag. Health Policy.

[201] Gardiner C., Weakley J., Burke L.M., Roach G.D., Sargent C., Maniar N., Townshend A., Halson S.L. (2023). The effect of caffeine on subsequent sleep: A systematic review and meta-analysis. Sleep Med. Rev..

[202] Gardiner C., Weakley J., Burke L.M., Roach G.D., Sargent C., Maniar N., Huynh M., Miller D.J., Townshend A., Halson S.L. (2025). The effect of alcohol on subsequent sleep in healthy adults: A systematic review and meta-analysis. Sleep Med. Rev..

[203] Barrea L., Pugliese G., Frias-Toral E., El Ghoch M., Castellucci B., Chapela S.P., Carignano M.d.L.A., Laudisio D., Savastano S., Colao A. (2023). Coffee consumption, health benefits and side effects: A narrative review and update for dietitians and nutritionists. Crit. Rev. Food Sci. Nutr..

[204] Silvani M.I., Werder R., Perret C. (2022). The influence of blue light on sleep, performance and wellbeing in young adults: A systematic review. Front. Physiol..

[205] AlShareef S.M. (2022). The impact of bedtime technology use on sleep quality and excessive daytime sleepiness in adults. Sleep Sci..

[206] Shen B., Ma C., Wu G., Liu H., Chen L., Yang G. (2023). Effects of exercise on circadian rhythms in humans. Front. Pharmacol..

[207] Lewis P., Korf H.W., Kuffer L., Groß J.V., Erren T.C. (2018). Exercise time cues (zeitgebers) for human circadian systems can foster health and improve performance: A systematic review. BMJ Open Sport Exerc. Med..

[208] Healy K.L., Morris A.R., Liu A.C. (2021). Circadian Synchrony: Sleep, Nutrition, and Physical Activity. Front. Netw. Physiol..

[209] Drăgoi C.M., Nicolae A.C., Ungurianu A., Margină D.M., Grădinaru D., Dumitrescu I.-B. (2024). Circadian Rhythms, Chrononutrition, Physical Training, and Redox Homeostasis-Molecular Mechanisms in Human Health. Cells.

[210] Kim N., Ka S., Park J. (2023). Effects of exercise timing and intensity on physiological circadian rhythm and sleep quality: A systematic review. Phys. Act. Nutr..

[211] Koch C.E., Leinweber B., Drengberg B.C., Blaum C., Oster H. (2017). Interaction between circadian rhythms and stress. Neurobiol. Stress.

[212] Blume C., Garbazza C., Spitschan M. (2019). Effects of light on human circadian rhythms, sleep and mood. Somnologie.

[213] Tähkämö L., Partonen T., Pesonen A.-K. (2019). Systematic review of light exposure impact on human circadian rhythm. Chronobiol. Int..

[214] Zabuliene L., Milionis C., Koukkou E., Ilias I. (2025). Exposure to artificial lighting at night: From an ecological challenge to a risk factor for glucose dysmetabolism and gestational diabetes? Narrative review. Ann. Med..

[215] Muscogiuri G., Poggiogalle E., Barrea L., Tarsitano M.G., Garifalos F., Liccardi A., Pugliese G., Savastano S., Colao A., Alviggi C. (2022). Exposure to artificial light at night: A common link for obesity and cancer?. Eur. J. Cancer.

[216] Cho Y., Ryu S.-H., Lee B.R., Kim K.H., Lee E., Choi J. (2015). Effects of artificial light at night on human health: A literature review of observational and experimental studies applied to exposure assessment. Chronobiol. Int..

[217] Davis L.K., Bumgarner J.R., Nelson R.J., Fonken L.K. (2023). Health Effects of Disrupted Circadian Rhythms by Artificial Light at Night. Policy Insights Behav. Brain Sci..

